# Mitigation potential of global ammonia emissions and related health impacts in the trade network

**DOI:** 10.1038/s41467-021-25854-3

**Published:** 2021-11-05

**Authors:** Rong Ma, Ke Li, Yixin Guo, Bo Zhang, Xueli Zhao, Soeren Linder, ChengHe Guan, Guoqian Chen, Yujie Gan, Jing Meng

**Affiliations:** 1 School of Economics and Management, Beihang University, Beijing, China; 2 Harvard–NUIST Joint Laboratory for Air Quality and Climate, Jiangsu Key Laboratory of Atmospheric Environment Monitoring and Pollution Control, Collaborative Innovation Center of Atmospheric Environment and Equipment Technology, School of Environmental Science and Engineering, Nanjing University of Information Science and Technology, Nanjing, China; 3 John A. Paulson School of Engineering and Applied Sciences, Harvard University, Cambridge, MA USA; 4 Princeton School of Public and International Affairs, Princeton University, Princeton, NJ USA; 5 School of Management, China University of Mining and Technology (Beijing), Beijing, China; 6 Joint Research Centre, Food Security Group, European Commissions, Ispra, Italy; 7 Arts and Science, New York University Shanghai, Shanghai, China; 8 Laboratory of Systems Ecology and Sustainability Science, College of Engineering, Peking University, Beijing, China; 9 School of Government, The Leo KoGuan Building, Peking University, 100871 Beijing, China; 10 The Bartlett School of Sustainable Construction, University of College London, London, WC1E 7HB UK; 11 Present Address: Laboratory for Climate and Ocean–Atmosphere Studies, Department of Atmospheric and Oceanic Sciences, School of Physics, Peking University, Beijing, China

**Keywords:** Atmospheric chemistry, Environmental impact, Environmental economics, Sustainability

## Abstract

Ammonia (NH_3_) emissions, mainly from agricultural sources, generate substantial health damage due to the adverse effects on air quality. NH_3_ emission reduction strategies are still far from being effective. In particular, a growing trade network in this era of globalization offers untapped emission mitigation potential that has been overlooked. Here we show that about one-fourth of global agricultural NH_3_ emissions in 2012 are trade-related. Globally they induce 61 thousand PM_2.5_-related premature mortalities, with 25 thousand deaths associated with crop cultivation and 36 thousand deaths with livestock production. The trade-related health damage network is regionally integrated and can be characterized by three trading communities. Thus, effective cooperation within trade-dependent communities will achieve considerable NH_3_ emission reductions allowed by technological advancements and trade structure adjustments. Identification of regional communities from network analysis offers a new perspective on addressing NH_3_ emissions and is also applicable to agricultural greenhouse gas emissions mitigation.

## Abstract (web summary)

Ammonia emissions from agricultural sources can cause severe health impacts. Here, the authors show that about 25% of global agricultural ammonia emissions in 2012 were related to international exported goods and caused 61 thousand PM2.5 related premature deaths, which points out large ammonia mitigation potential in international trade.

## Introduction

With air pollution being reduced globally by controlling pollutants from industrial sectors, the far-less-regulated ammonia (NH_3_) emissions consequently become an important driver for fine particulate matter (PM_2.5_) pollution^1–4^. NH_3_ emissions contribute to PM_2.5_ pollution through the chemical formation of particulate ammonium sulfate and ammonium nitrate^4,5^ and lead to tens of thousands of deaths annually^6^. Nearly 90% of global NH_3_ emissions are emitted from agricultural sources^1^, including ammonia-based fertilizers and animal manure. Unfortunately, regulations for agricultural NH_3_ emissions are overall ineffective worldwide^1^. Outpacing many industrial sectors, agriculture is the leading sector in driving anthropogenic PM_2.5_ pollution in Europe and the eastern USA^4,6,7^. NH_3_ emissions are currently not regulated over high NH_3_ emitting regions, e.g., China^1^, although recent research shows that improving agricultural nitrogen management can achieve 34% reductions and reduce PM_2.5_ by up to 8 μg m^−3^ (ref. ^8^) More importantly, future increases in agricultural production to accommodate food demand of a growing population will increase the health risks from NH_3_-related environmental consequences^9,10^. As such, developing strategies to reduce NH_3_ emissions is urgent and would generate substantial environmental and health benefits^1,6,8,11^.

Substantial efforts have already been made to reduce pollutant emissions at local scales^1,8^. In a globalized world, however, localized agricultural production is increasingly connected to foreign consumption owing to the expanding agricultural trade in order to meet food and nutritional demands around the world^12^. The current trade volume of global agricultural commodities accounts for over 20% of global agricultural production^13^, mostly occurring between Organisation for Economic Cooperation and Development (OECD) and non-OECD countries (such as China, India, and other Asian countries). Substantial NH_3_ emissions are related to international exports of agricultural commodities by mostly developing countries to meet the growing food demand of the developed world^14^. Understanding NH_3_ emissions embodied in international trade offers considerable potential to abate NH_3_ emissions.

NH_3_ emission transfers through the global trade network can be quantified by global multiregional input–output (MRIO) models, which have been applied to measure trade-induced emissions of greenhouse gases^15–18^, primary PM_2.5_, and secondary PM_2.5_ precursors^19–25^. Oita et al.^14^ reported that about 26% of NH_3_ emissions in 2010 were embodied in the international trade of commodities. However, little attention was paid to the related public health burden, except for several recent analyses on the health impact of trade-related primary PM_2.5_^23–25^ and secondary PM_2.5_ precursors^22^. Although these studies shed light on the international dimension of consumption-driven environmental pollution and related health risks, their insights into each type of air pollutant have been counteracted because of the different sources and mitigation potential. Especially, previous studies focused mainly on pollutant emissions from industrial sectors, and agricultural NH_3_ emission transfers and their environmental and health outcomes are still not fully understood^1^.

Furthermore, transfers of the health burdens from trade-related NH_3_ emissions are determined by the structure of the international trade of agricultural commodities. Comparative advantages, such as availability of arable land, water resources, technologies, and geographical location, prompt various economies to participate in the production, processing, and trade of agricultural commodities. Those interregional activities transfer NH_3_ emissions and their health outcomes, together weaving a complex network^26^. Unveiling the network characteristics of health-effect transfers can target important regions, production sectors, consumption categories, and communities for reducing NH_3_ emissions and mitigating health damages.

In this work, we aim to explore the mitigation potential of global ammonia emissions by analyzing the role of the international trade network. We show the trade-induced global agricultural NH_3_ emissions, consequent PM_2.5_ formation and related health impacts of the year 2012 in 181 economies, demonstrating large NH_3_ mitigation potential in international trade and associated benefits. We identify the role of leading communities in transferring the health impacts through international trade. We further demonstrate the potential of technological advancements and trade structure adjustments within leading countries in reducing trade-related NH_3_ emissions. These findings point out the importance of international collaborative efforts for the formulation of comprehensive international environmental policies and actions for addressing NH_3_ that are overlooked.

## Results

### Health burdens of trade-related NH_3_ emissions

**Fig. 1:**
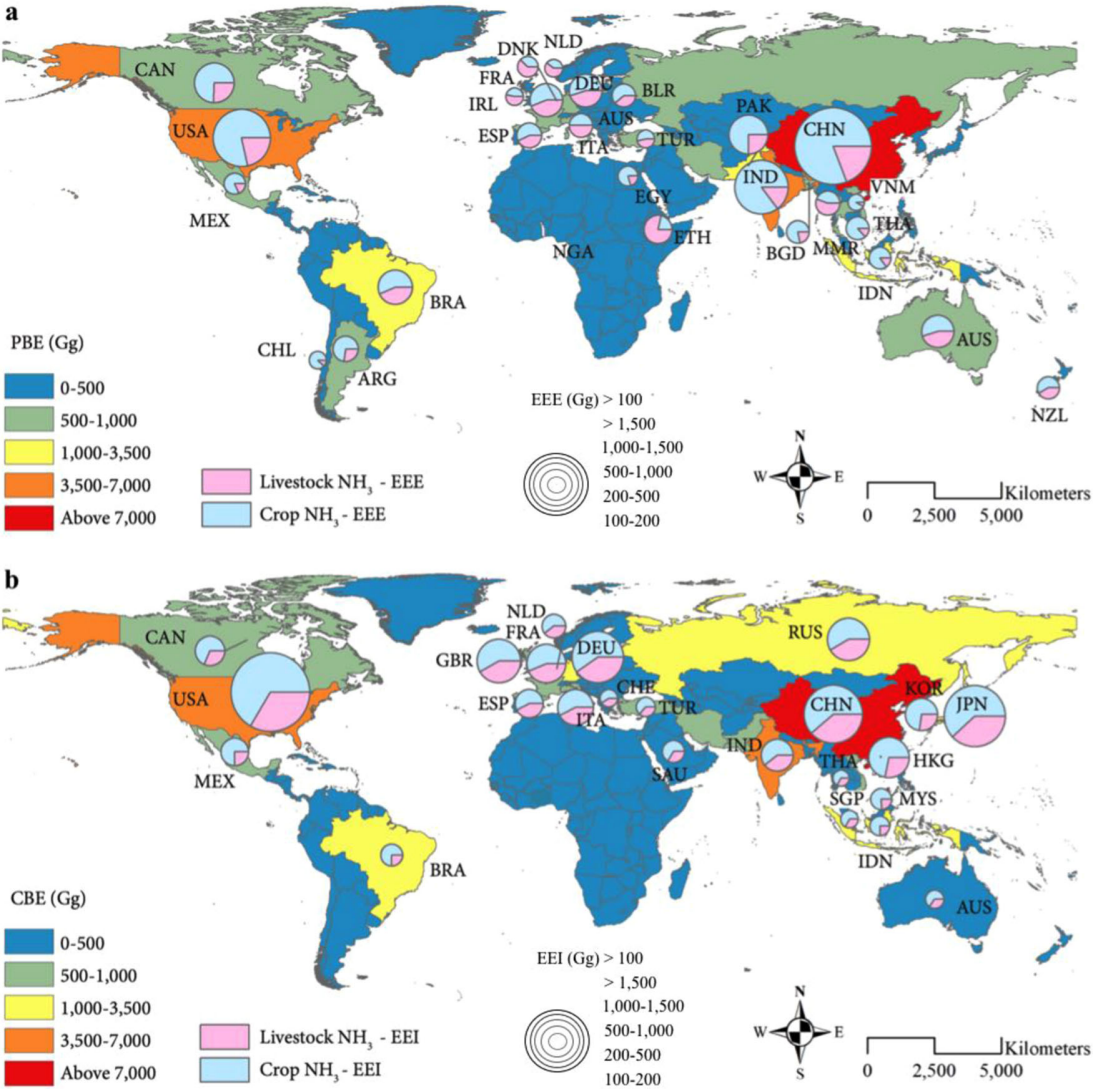
Global agricultural NH_3_ emissions associated with production, consumption, and trade. **a** Production-based emissions (PBEs) of NH_3_ (shaded) and export-related emissions (EEEs) of NH_3_ (pie charts) (Gg) in 2012. Pie charts inserted in (**a**) are the countries (highlighted by country’s abbreviation) with high EEE NH_3_ emissions from livestock and crop cultivation, respectively. **b** Consumption-based (CBE) and import-related emissions (EEIs) of NH_3_ (Gg) in 2012. Detailed results for each country are provided in the Supplementary Data files. The three-letter country abbreviations inserted in the plot are detailed in Supplementary Data 6. Maps were created by using ArcGIS version 10.7.1 (ESRI https://www.esri.com/en-us/arcgis/about-arcgis/overview).

Global trade-induced NH_3_ emissions of the year 2012 are assessed using the MRIO model with detailed NH_3_ emissions estimates from the Emissions Database for Global Atmospheric Research (EDGAR v4.3.2) inventory^27^ (see “Methods”). Since agricultural NH_3_ emissions (52,325 Gg) account for 89% of the global NH_3_ emissions (58,671 Gg) in 2012, we focus on NH_3_ emissions from the agricultural sector in this study. The embodied NH_3_ emissions in international trade balance (EEB) can be obtained as the difference of import-related emissions (EEI, total emissions in other regions related to domestic consumption) and export-related emissions (EEE, the total domestic emissions related to final consumption in other regions) (see Fig. 1 and “Methods”). An economy with a positive value of EEB is a net importer of embodied NH_3_ emissions, while that with a negative EEB is a net exporter. By linking the local emissions to global consumption, an estimated 23% (11,840 Gg) of global agricultural production-based emissions (PBEs), namely emissions caused by domestic production, are associated with international exports (Fig. 1a and Supplementary Data 1). Our estimation is consistent with the previously reported 26% of NH_3_ emissions embodied in the international trade in 2010^14^. Owing to such substantial agricultural NH_3_ emissions embodied in international trade, the PBE of NH_3_ in most economies are remarkably different from their consumption-based emissions (CBEs), which allocate emissions occurring during food production and distribution to final consumers (Fig. 1b and Supplementary Data 2). It thus means that global transfers of agricultural NH_3_ emissions (Supplementary Data 3 and Supplementary Table 1) can reallocate PM_2.5_ and public health burdens across borders, i.e., improving (harming) air quality and health in importing (exporting) countries.

**Fig. 2:**
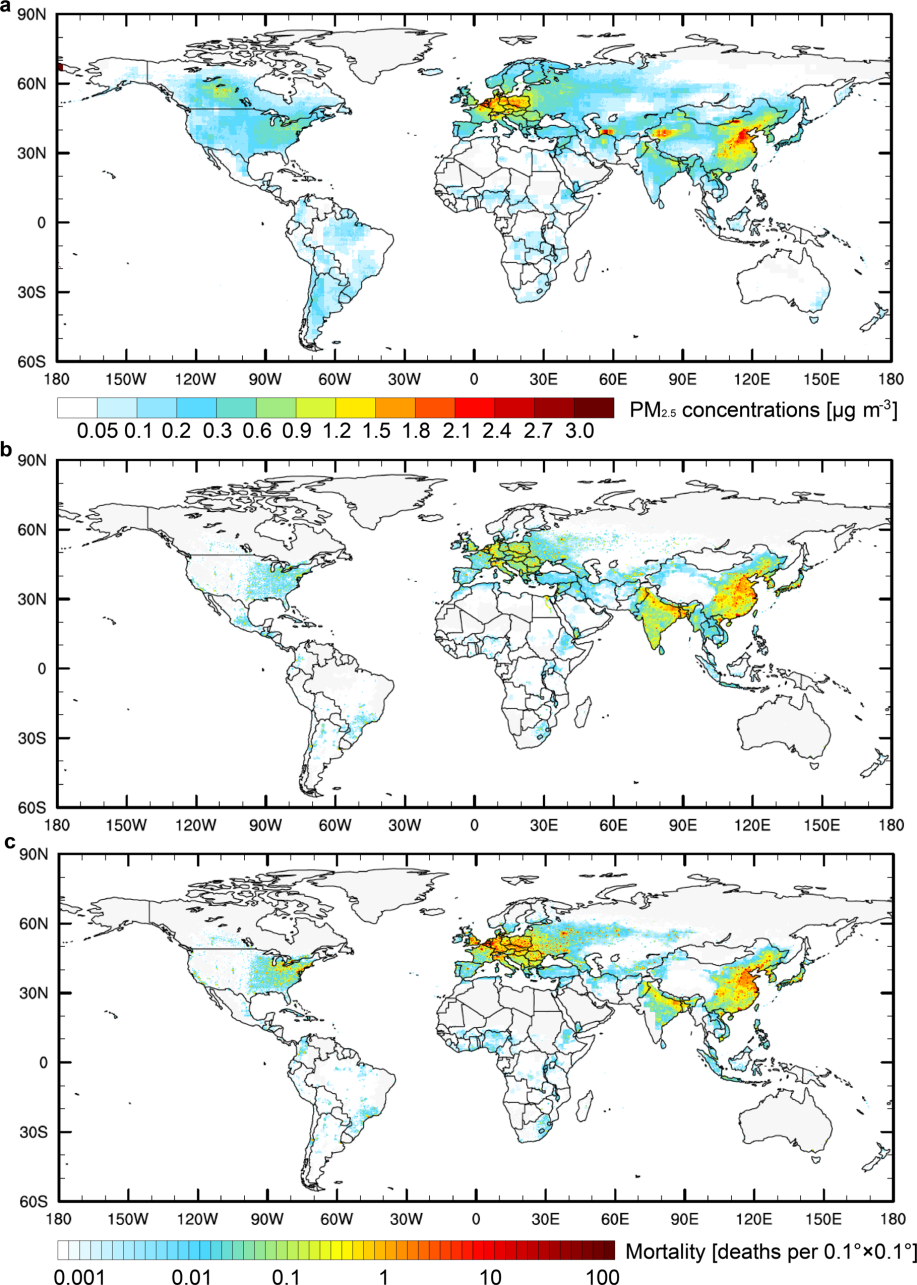
Air quality and health impacts of export-related NH_3_ emissions in 2012. **a** PM_2.5_ concentrations (μg m^−3^) induced by export-related NH_3_ emissions in 2012 are calculated by GEOS-Chem simulations. Attributable premature mortality density (deaths per 0.1° × 0.1° a^−1^) due to export-related NH_3_ emissions from **b** crop production and **c** livestock production. The attributable premature mortality is determined by GEOS-Chem modeled fractional contributions of export-driven NH_3_ emissions to total PM_2.5_ and the calibrated high-resolution PM_2.5_ data from GBD 2013^26^. Premature mortality on a resolution of 0.1° × 0.1° is estimated following the methods of the GBD study to estimate the premature deaths from ambient PM_2.5_ exposure (see “Methods”). Maps were created by using the NCAR Command Language, version 6.4.0 (NCAR, 10.5065/D6WD3XH5).

We quantify the contribution of trade-related NH_3_ emissions to PM_2.5_ exposure by utilizing a global chemical transport model (CTM) (GEOS-Chem) by perturbing NH_3_ emissions embodied in exported products (export-related emissions) for 181 countries (see “Methods”). Figure 2a shows that NH_3_ emissions resulting from producing final products that are ultimately consumed abroad occur in many developing countries, with adverse effects on local air quality. About 1–2 μg m^−3^ of PM_2.5_ in eastern China is contributed by agricultural NH_3_ emitted during the production of food that is exported. We found a similar magnitude of contributions to local PM_2.5_ for export in other countries, i.e., 0.6–1.2 μg m^−3^ in northern India and Pakistan, 0.6–1.5 μg m^−3^ in northern Italy and eastern European countries (e.g., Poland, Belarus, Ukraine), and 0.3–0.9 μg m^−3^ in the eastern USA and central Canada.

The associated public health burden is estimated using the integrated exposure–response (IER) functions following the method of the Global Burden of Disease (GBD) study^28^ (see “Methods”). The estimated premature deaths attributed to ambient PM_2.5_ exposure is a function of export-related NH_3_ emissions, local PM_2.5_ levels, population densities, and baseline mortality for different diseases. Here we consider the impacts from the four leading causes of death: ischemic heart disease, chronic obstructive pulmonary disease, cerebrovascular disease, and lung cancer. We estimated the mortality contribution from sectoral export-related agricultural NH_3_ emissions based on an assumption that the contribution of one sector to the disease burden of PM_2.5_ is directly proportional to its share of PM_2.5_ concentration.

For a given country, the premature deaths from its sectoral export-related NH_3_ emissions can be calculated by multiplying its fractional contribution of sectoral export-related NH_3_ emissions to PM_2.5_ concentration by the total PM_2.5_ concentration-related mortalities for each 0.1° × 0.1° grid cell. The fractional contribution of sectoral export-related NH_3_ emissions to PM_2.5_ was estimated by the GEOS-Chem simulations (see “Methods”). The export-related NH_3_ emissions are related to 61 thousand premature deaths, especially in many developing countries (Supplementary Fig. 1 and Supplementary Table 2). High premature mortality is found in China (26.3 thousand deaths) and India (6.2 thousand deaths), due to their higher PM_2.5_ concentrations from export-related agricultural NH_3_ emissions and population densities. In Southeast Asia, premature mortality is estimated at about 2.0 thousand deaths, of which Bangladesh and Vietnam account for ~45% (0.9 thousand deaths) and 32% (0.7 thousand deaths). In Pakistan, ~37% of agricultural NH_3_ emissions and 0.9 thousand deaths are related to exports. PM_2.5_ pollution from export-related NH_3_ emissions is responsible for 2.1 thousand deaths in the USA. In Europe, the estimated premature mortality in Eastern European countries (9.7 thousand deaths) is much higher than those in Western Europe (3.9 thousand deaths).

Figure 2b, c shows the health burdens estimated from sectoral export-related NH_3_ emissions. Premature mortality induced by export-related livestock production is 36 thousand deaths, and by export-related crop production is 25 thousand deaths. It suggests that NH_3_ emissions from trade-related livestock production need more strict control due to its higher overlap with residential regions that are populated and have high emissions of NO_*x*_ and SO_2_, particularly over Mainland China and India (Fig. 2). Supplementary Table 3 shows the top 20 trading pairs of sectoral NH_3_ trade-related health impacts. We found that Mainland China and India suffer substantial health costs via exporting to developed countries. Moreover, there are large variations in the health effects across different regions of each country due to differences in local PM_2.5_ levels, population densities and agricultural production activities. For example, 73% (90%) of health burden from crop sector (livestock sector) in the USA was found on eastern USA (east of 95°W), and 58% (78%) of health burden from crop sector (livestock sector) was concentrated in northern India (north of 24°N). Northern China (north of 30°N) is the hotspot of China’s related premature deaths, accounting for about 70% from the crop sector or livestock sector. These results suggest that the health effects related to the livestock sector are more likely to be regionally concentrated, so place-based strategies on regional emission reduction are particularly needed. Regions need to consider more strict regulations on emissions from the sector that causes larger health burdens.

Overall, health impacts related to export-related emissions from major developing countries such as China, India, Pakistan, and Southeastern Asian countries account for ~70% of worldwide premature mortality. This highlights the huge potentials and benefits of reducing NH_3_ emissions in international trade.

### Structure of NH_3_ trade-related health-effect network

Using embodied emissions in export and import, we decompose the country-level health burdens into bilateral health effects and construct an interconnected network of NH_3_ trade-related health impacts. Unlike previous studies^14^ that explore international trade relationships from the perspective of emissions only, here we conduct a more comprehensive analysis by linking the emission-induced health effects to trade networks. The health-effect network is not only determined by export-related emissions but also by local air pollution levels and population densities. Identifying this network structure can help trace the origins of the health impacts and pinpoint effective mitigation strategies of NH_3_ emissions embodied in international trade.

**Fig. 3:**
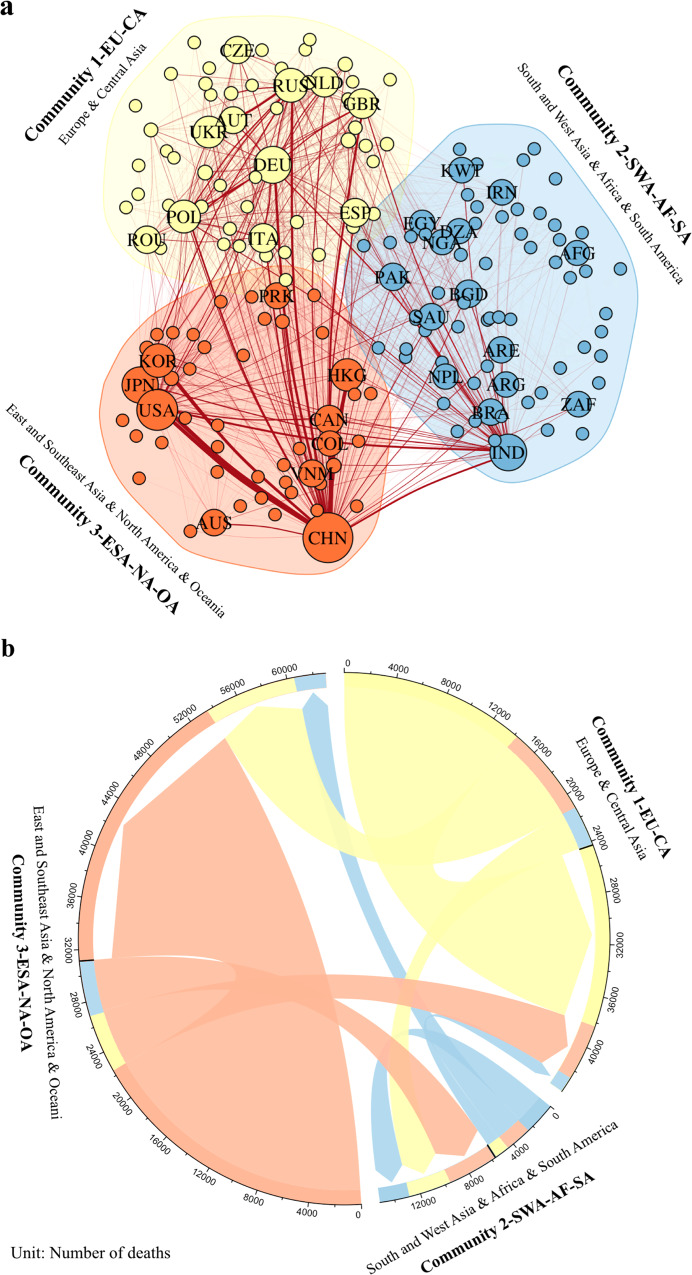
Regional communities of NH_3_ trade-related health-effect network. **a** The partitions of communities. Along with each community, the major hub economies are also indicated. The size of a circle represents the relative trade-related health loss. The width of a connecting line between two circles represents the relative health loss attributed to the trade between the two nodes. **b** The intracommunity and intercommunity health-effect flows (number of deaths). Community 1-EU-CA (in yellow) is mainly formed by countries in Europe and Central Asia; Community 2-SWA-AF-SA (in blue) consists of countries in South and West Asia, Africa, and South America; Community 3-ESA-NA-OA (in orange) is dominated by countries in East and Southeast Asia, North America and Oceania. The width of the connecting line represents trade-related health loss. The three-letter country abbreviations inserted in the plot are detailed in Supplementary Data 6. Supplementary Fig. 6 shows the geographical distributions of communities.

Figure 3 shows that the global health-effect network is characterized by three trading communities. Community 1-EU-CA mainly consists of countries in Europe and Central Asia. Community 2-SWA-AF-SA is formed by countries in South and West Asia, Africa, and South America, while Community 3-ESA-NA-OA is dominated by East and Southeast Asia, North America, and Oceania. Countries within each community are closely integrated and can be extrinsically motivated to cooperate on emission reduction. We employ the Girvan–Newman community detection algorithm^29^ to reveal the clustering features (see “Methods”). The community structure is mainly regulated by geographic proximity, which is the pivotal determinant of international agricultural trade, partly due to the low value-added but high transportation and storage costs of agricultural products. Overall, 64% of the total trade-related health effects are attributed to intracommunity flows, coinciding with the high regional integration of agricultural goods trade. More specifically, in all communities, the major portion of export-related health effects are attributed to themselves. The import-related health effects of all communities are also sourced from themselves, except for Community 2-SWA-AF-SA. This regional amalgamation implies that the priority of reducing trade-induced NH_3_ emissions needs to be given to the mitigation within each community. Despite the common feature in clustering, the roles of the three communities vary in the network. More than 75% of import-related health effects in Community 2-SWA-AF-SA are from other communities, highlighting its role as an importer of agricultural products. On the other hand, only 28% of export-related health effects in Community 3-ESA-NA-OA are induced by other communities despite that it has the greatest import-related health effects. This indicates the high mitigation responsibilities of Community 3-ESA-NA-OA are largely attributed to itself, while Community 2-SWA-AF-SA needs to undertake more obligations than what its direct emissions suggest.

In each community, some hub countries connect the loosely linked economies. To evaluate the network’s heterogeneity, we examine degree distributions of the network (Supplementary Fig. 2). The network approximately follows a scale-free network^30^ that is stable against random failures but vulnerable to targeted attacks^31^ (see “Methods”). This means effectively mitigating the health effects from trade-related NH_3_ emissions requires more effort given to the hub economies because changing their trade practices and emission intensities per gross output will generate profound influences on the whole network.

We further exploit network indicators to identify hub economies (see “Methods”). Mainland China, USA, India, and Germany play the most vital roles of main bridges in their communities, respectively (Supplementary Fig. 3c, d), based on the betweenness centrality that assesses node connectivity and intermediacy^32^. Although the number of importing sources (in-degree) of major European economies in Community 1-EU-CA are greater than Japan and Hong Kong, their import-related health effects are noticeably smaller (Supplementary Fig. 3a). By contrast, China and India, the leading countries in Community 3-ESA-NA-OA and Community 2-SWA-AF-SA, bear more than 50% of aggregated export-related health loss (Supplementary Fig. 3b) and share a similar number of exporting destinations (out-degree). As reflected by eigenvector centrality, Japan and Hong Kong also act as important bridges since they are major importers of agricultural products in their communities (Supplementary Fig. 3e). Lastly, small-degree economies tend to trade with hub economies, as indicated by Supplementary Fig. 3f. It suggests that the health-effect network displays high levels of regional integration despite some heterogeneity. This regionally clustered trade network offers potential solutions for NH_3_ emission reduction through regional cooperation within each community.

### Implications for NH_3_ emission mitigation and international cooperation

Both regional and global perspectives are essential to developing NH_3_ emission reduction strategies. The identification of regional communities from the network analysis provides a new lens on NH_3_ emission control. Analogous to “climate clubs” proposed by Nordhaus^33^, countries identified in the same community by our methods are tightly connected in the health-effect network, so they are strongly incentivized to form a mitigation club of NH_3_ emissions, i.e., club members are expected to promote technological advancements and trade structure adjustments to obtain overall NH_3_ emission reductions within each community. The integrated structure also suggests that fewer trade barriers exist within the community and that member states are more likely to collaborate. Importing countries will directly benefit from the adjustments due to less pollution spillover. Countries with excessive exports will reduce health damages but also sacrifice their economic gains from exports. To align their incentives, intergovernmental coordination, as highlighted by the Interconvention Nitrogen Coordination Mechanism^34^, is a requisite for member countries to form a mitigation “club.” Members in violation of club rules will be penalized by uniformly punitive tariffs and other multilateral policies. As long as the mutual gains from emission reduction are sufficiently large, countries are motivated to obey club rules^33^. Here we propose NH_3_ reduction scenarios through trade structure adjustments and technological advancements within the regionally trade-dependent communities (Table 1).

**Table 1:** NH_3_ emissions reductions (unit: Gg) achieved through trade-side, consumption-side, and production-side strategies for the three communities.

Regions	Community	Community	Community
	1-EU-CA	2-SWA-AF-SA	3-ESA-NA-OA
Baseline NH_3_ emissions	8700	16,600	27,100
Trade-side strategies			
Import substitution	210	50	80
Export transfer	750	450	470
Production-side strategies			
Reducing overuse of N in grain crops	550	1400	2500
Deep fertilizer placement	1700	3800	6500
Use enhanced-efficiency fertilizers	1600	3700	6300
Moderate manure management improvements	1400	1600	2400
Drastic manure management improvements	2700	3200	4800
Maximum technically feasible reductions (MTFR)	2800	7100	15,200
Consumption-side strategies			
Eliminating food waste and loss	2300	4500	7800
Reducing beef consumption by 20%	260	380	570
Reducing beef consumption by 50%	650	940	1400

Community 1-EU-CA is formed by countries in Europe and Central Asia; Community 2-SWA-AF-SA consists of countries in South and West Asia, Africa, and South America; Community 3-ESA-NA-OA is dominated by countries in East and Southeast Asia, North America, and Oceania. The scenario of “Reducing overuse of N in grain crops” has no harm to grain crop yields according to Mueller et al.^61^. The two “manure management improvements” scenarios include various manure handling technologies that can reduce NH_3_ emissions from animal manure by 30–90% (see “Methods” section for the design of moderate and drastic manure management improvement scenarios). The “MTFR” scenario by 2050 is calculated from the GAINS (Greenhouse gas-Air pollution Interactions and Synergies) model^36^.

Trade structure adjustments include the import substitution scenario and export transfer scenario. In the scenario of import substitution, we consider replacing imports of agricultural goods with domestic production if the emission intensity of the exporter is higher than the importer. Larger emission reductions will be achieved if production is allowed to be transferred to a third country with emission intensities even lower than both the original exporter and importer. The export transfer scenario thus will minimize the agricultural trade-related NH_3_ emissions by taking total exports as given and reorganizing the production structure of exported goods. Nevertheless, complete substitution is unrealistic. Countries are unable to expand their agricultural production beyond their total capacity. We impose this constraint with their potential capacity in agricultural production, measured by the area of arable land multiplied by the value of agricultural products per unit area. We also limit trade substitution to the countries with similar annual average temperature and precipitation (see “Methods”).

This scenario analysis of import substitution and export transfer is applied to countries within each community. Table 1 shows that import substitution has modest effects on emission reduction, but the export transfer displays considerable potentials to reduce NH_3_ emissions. Especially, export transfer in both Community 1-EU-CA could reduce NH_3_ emissions by 750 Gg. With NH_3_ emission reduction of over 100 Gg, Belarus, Germany, France, and Spain are the most benefited countries in Community 1-EU-CA (Supplementary Data 4). In Community 3-ESA-NA-OA, NH_3_ emissions are reduced by 230 Gg in Mainland China, followed by Myanmar (80 Gg), Mexico (70 Gg), and Chile (60 Gg). The most benefited countries in Community 2-SWA-AF-SA are Ethiopia (150 Gg), Pakistan (100 Gg), and Argentina (80 Gg). The contribution of import substitution to NH_3_ reduction is largest in Community 1-EU-CA (210 Gg) (Supplementary Data 5). This estimated NH_3_ emission reduction demonstrates that regional efforts within each community through trade structure adjustments have substantial potential in NH_3_ emission mitigation even under current production efficiency. The distinction between the scenarios of import substitution and export transfer further implies that effective trade adjustments to reduce NH_3_ emissions call for community-level multilateral cooperation instead of unilateral trade substitution.

Technological advancement is expected to be the most effective way to mitigate NH_3_ emissions^1,35^. Here the regionally integrated health-effect network incentivizes countries within the same community to foster technological advancements in reducing NH_3_ emissions. We estimate potential global NH_3_ emission reduction achieved through the production side and consumption side (see “Methods”). The effectiveness of food production and consumption strategies in reducing NH_3_ emissions are summarized in Table 1. In terms of production-side mitigation, we consider three improved management of agricultural nitrogen from crop production, and two scenarios from livestock production. These technological advancements display huge potentials to reduce NH_3_ emissions in Table 1. Especially, strict mitigations (e.g., deep fertilizer placement or use of enhanced-efficiency fertilizers) for crop production show substantial potentials for Community 2-SWA-AF-SA and Community 3-ESA-NA-OA where NH_3_ emissions are high. For comparison, we also show the maximum technically feasible reduction (MTFR) scenario by the year 2050 calculated from the GAINS (Greenhouse gas-Air pollution Interactions and Synergies) model^36^. The MTFR scenario implements the best available mitigation technology that varies regionally. The strictest NH_3_ emission reduction (e.g., deep fertilizer placement and drastic manure management) we propose is comparable with the NH_3_ emission reduction under the MTFR scenario by the year 2050.

In terms of consumption-side mitigation, we consider dietary adjustment scenarios in Table 1. Given current food production and consumption practices worldwide, different regions should prioritize different strategies. USA and Europe, which already have developed modern agriculture with reasonably good nitrogen management practices, could focus on consumption-side strategies. Developing countries in Community 2-SWA-AF-SA and Community 3-ESA-NA-OA, such as India and Mainland China need to dedicate themselves to both production-side and consumption-side strategies.

To further estimate the health benefits of NH_3_ emission reductions under different scenarios, we select three scenarios (i.e., export transfer, reducing overuse of N in grain crops, and reducing beef consumption by 20%) for quantifying the associated PM_2.5_ changes and related health effects by conducting GEOS-Chem simulations. Under each scenario, we applied the reduction ratio to spatiotemporally-changed NH_3_ emissions for each country. It is also assumed that population densities and baseline mortality in IER functions are the same as in the year 2012. The estimated health benefits under these three scenarios are listed in Supplementary Table 4. Overall, we find that the NH_3_ emission reduction by trade-side strategies is comparable to the reduction by consumption-side strategies, while production-side strategies tend to be the dominant driver for future NH_3_ emission reduction.

Given the absence of NH_3_ emission regulations, the Fourth United Nations Environment Assembly in March 2019 approved the first-ever global resolution on nitrogen^37^, and intended to establish an intergovernmental coordination mechanism on nitrogen policies. However, it is not yet clear how long it will take for countries to reach a binding agreement and how ambitious the mitigation target will be. The regional communities identified by our network analyses and the community-based NH_3_ emission controls proposed in this research will fill this gap. Countries in the same community are strongly incentivized to form an NH_3_ mitigation club to promote technological advancements and trade structure adjustments to obtain overall NH_3_ emission reduction within each community.

## Discussion

In summary, interventions on interregional and intraregional trade structures in each community contribute to significant NH_3_ emission reduction, due to huge health impacts from trade-induced global agricultural NH_3_ emissions. Countries are incentivized to optimize trade structure and consume fewer emission-intensive agricultural products, while this could, in turn, prompt upstream suppliers to adopt environmentally benign agricultural technologies and minimize the negative environmental outcomes of agricultural production. Our results demonstrate that there are appreciable potentials in reducing NH_3_ emissions through effective cooperation within the regional trade-dependent community. Naturally, the proposed solution in this study is also applicable to the mitigation of agricultural greenhouse gas emissions.

Some limitations exist in this study, i.e. uncertainties in NH_3_ emission inventory, de-coupled approach for reducing NH_3_ from crop and livestock sectors, and assumptions with trade scenarios. For NH_3_ emission inventory, the low resolution of agricultural sectors in many developing countries could cause uncertainty to the global MRIO analysis. More efforts are required to compile reliable and high-resolution NH_3_ emission inventories associated with disaggregated agricultural sectors.

For our evaluation of ammonia emission mitigation potential, we have treated crop and livestock sectors as separate and independent sectoral sources. In reality, they are interconnected subcomponents of agricultural systems^38^. In detail, our analyses of the effects of manure deep placement (Table 1) assume that abundant cropland exists for manure application and that crop farmers will reduce the application of inorganic nitrogenous fertilizers accordingly to avoid excess N use. These assumptions create real-world logistical challenges. For example, animal production has become more concentrated in the USA. In many states, the amount of manure excreted has exceeded local assimilative cropland capacity, demanding off-farm export^39^. Farmers may not reduce inorganic fertilizer N use without governmental education efforts or economic/policy mechanisms that avoid excess N use^40^. In addition, manure acidification, although reduces NH_3_ emissions during storage, may result in a larger amount of N susceptible for NH_3_ losses during the disposal stage. Thus, future work using integrated models that represent N flows in both crop and livestock sectors and their interactions may provide a more precise estimation of NH_3_ emission responses to sectoral strategies.

For trade reduction scenarios, other factors, such as political interests, also have determinant effects on international trade. However, in the past decades, countries have resolved many transnational conflicts and spillovers through international agreements. For example, the international collaboration on carbon emissions moves fast, which includes some trade-related measures such as imposing the carbon tax. The evidence of mitigation potential comes first, and then we can look forward to the effect. Therefore, we believe that the trade-side strategies, as well as other joined-up efforts proposed in the Interconvention Nitrogen Coordination Mechanism^34^, could be of interest for future international cooperation.

## Methods

### NH_3_ emissions

Agricultural sources of NH_3_ emissions refer to manure management, direct and indirect soil emissions, manure in pasture/range/paddock, and agricultural waste burning. The estimation of agricultural NH_3_ emissions at the country level is extremely challenging, due to the fact that a large amount of activity-level data and emission factors are hard to obtain. The EDGAR v4.3.2 emission database^27^ from Joint Research Centre, European Commission, has updated the bottom-up inventories of NH_3_ emissions of nations to the year of 2012, which make it possible for a more systematic study on consumption-based accountings of global NH_3_ emissions.

### Global MRIO model

The global MRIO tables covering multiple regions of the world have been prepared by multiple organizations. Among these tables, the MRIO tables from the Eora database cover the most regions^41,42^, which have been widely used to analyze the embodied resource and environmental elements in international trade^43–45^. In this study, the Eora database is adopted to build the global MRIO table for 2012.

The global MRIO model, incorporating direct emission inventories, reveals the NH_3_ emissions induced by final demand and international trade. MRIO can trace the emissions back to the original source that produced the emissions even if products were intermediate constituents in a multiregional supply chain. To perform the MRIO modeling, we should extract the direct emission data that are related to economic activities and reallocate these data to each industrial sector of different economies. The resulting emissions at the sectorial level are used in the MRIO model to link the emissions to consumption and trade. Assume that the number of sectors of country *s* is ${k}_{s}$, the number of countries is *n*, and denote $N=\mathop{\sum }\nolimits_{s=1}^{n}{k}_{s}$. According to the balance of the global MRIO model, the basic linear equation can be expressed as

1 $${{{{{\bf{X}}}}}}={{{{{\rm{A}}}}}}{{{{{\bf{X}}}}}}+{{{{{\bf{F}}}}}}$$

where ${{{{{\bf{X}}}}}}$ is the $N\times 1$ gross-output vector, ${{{{{\rm{A}}}}}}$ is the $N\times N$ technical coefficient matrix, and ${{{{{\bf{F}}}}}}$ stands for the $N\times 1$ final-consumption vector.

After that, the Leontief inverse matrix can be obtained from Eq. (1):

2 $$L={(I-A)}^{-1}$$

Here, ${(I-A)}^{-1}$ is the Leontief inverse matrix, which shows the total production of each sector required to satisfy the final demand in the region; $I$ is the identity matrix. $D$ refers to the $N\times N$matrix diagonalized from the $N\times 1$ vector of sectorial NH_3_ emission intensities (NH_3_ emissions per output). Since we are interested in the embodied agricultural NH_3_ emissions in global trade, the emission intensities of nonagricultural sectors are assigned to be zero. The global NH_3_ emission flow matrix can be acquired by

3 $$C=DL\hat{F}$$

where $\hat{F}$ is a $N\times N$ matrix diagonalized from the vector ${{{{{\bf{F}}}}}}$. The NH_3_ emission flow matrix $C$ can be written as the following block matrix4where ${C}_{st}$ is a ${k}_{s}\times {k}_{t}$ matrix. When *s* *≠* *t*, ${C}_{st}$ denotes the emissions produced in region *s* that are related to the final consumption of region *t*. When *s* *=* *t*, ${C}_{st}$ represents emissions related to final consumption produced locally. Let ${T}_{st}$ refer to the sum of each element ${C}_{st}^{(ij)}$ in ${C}_{st}$

5 $${T}_{st}=\mathop{\sum }\limits_{i\,=\,1}^{{k}_{s}}\mathop{\sum }\limits_{j\,=\,1}^{{k}_{t}}{C}_{st}^{(ij)}$$

${T}_{st}$ is a scalar that represents the total embodied agricultural emissions that are produced in region *s* and related to the final consumption of region *t*.

Based on the NH_3_ flows matrix, two key indicators that reflect the impacts of international trade on NH_3_ emissions can be deduced. The NH_3_ emissions embodied in international import and export are expressed as

6 $${{{{{\mathrm{EE}}}}}}{{{{{\mathrm{I}}}}}}^{s}=\mathop{\sum }\limits_{t=1,t\ne s}^{n}{T}_{ts}$$

7 $${{{{{\mathrm{EE}}}}}}{{{{{\mathrm{E}}}}}}^{s}=\mathop{\sum }\limits_{t=1,t\ne s}^{n}{T}_{st}$$

8 $${{{{{\mathrm{EE}}}}}}{{{{{\mathrm{B}}}}}}^{s}={{{{{\mathrm{EE}}}}}}{{{{{\mathrm{I}}}}}}^{s}-{{{{{\mathrm{EE}}}}}}{{{{{\mathrm{E}}}}}}^{s}$$

where ${{{{{\mathrm{EE}}}}}}{{{{{\mathrm{I}}}}}}^{s}$ is the total emissions in other regions related to consumption in region *s*, while ${{{{{\mathrm{EE}}}}}}{{{{{\mathrm{E}}}}}}^{s}$ is the total emissions in region *s* related to final consumption in other regions. The embodied NH_3_ emissions in international trade balance (EEB) can be obtained as the difference of import (EEI) and export (EEE). The EEB is also equal to the difference between the CBE emissions and its PBE emissions of a region. A positive value of EEB means that a region’s CBE emissions are larger than its PBE emissions.

### Adjustment to sectors and regions

The 189 countries/regions in the original MRIO table are merged with the EDGAR database. Serbia and Montenegro are merged into one country. Seven regions cannot be merged with the EDGAR database and thereby are dropped (Andorra, Former USSR, Gaza Strip, Liechtenstein, Monaco, San Marino, South Sudan). Finally, we have 181 economies (Supplementary Data 6) and 14,839 economy–sector pairs.

In the Full Eora database, most countries have more than two agricultural sectors. Crop and livestock sectors differ noticeably in their NH_3_ emission intensities (emissions per output). Therefore, we allocate NH_3_ emissions to crop and livestock sectors, respectively. More specifically, for each region, we assume the same emission intensity (emission per output) for different crop sectors, and allocate emissions to the detailed sectors based on their outputs. Similarly, we assume the same emission intensity for different livestock sectors and allocate emissions according to their outputs.

Furthermore, input–output analysis is susceptible to aggregation errors due to coarse sector classifications. For example, the ratio of export-related emissions to PBEs in Ethiopia is more than 90%, because it exports the agricultural products with smaller embodied emissions (such as coffee)^14^. Even though we use the Full Eora MRIO database that covers a comprehensive set of sectors for developed countries, some developing countries are only recorded in 26 sectors. To alleviate potential aggregation biases, we use product-level trade information from the United Nations Comtrade Database and follow Oita et al.^14^ to manually correct misallocation for countries susceptible to aggregation errors. Supplementary Table 5 shows the countries and exported commodities to be adjusted for aggregation errors. Due to missing data in the Comtrade database, we do not adjust exports of five small countries (Bermuda, Brunei Darussalam, Cape Verde, Cayman Islands, Netherlands Antilles) that are considered by Oita et al.^14^ We adjust exports of another country (Papua New Guinea) that is also susceptible to aggregation errors due to large export of palm oil, coffee, and cocoa beans. The embodied emissions calculated with the Eora-26 MRIO database, which has coarser sector classifications, are quite close to the results by the Full Eora database with adjustment to aggregation errors (Supplementary Fig. 4). This suggests that potential aggregation errors have a limited effect on the calculations and are unlikely to bias the analysis.

### Complex network model

We use complex network indicators and the community detection method to analyze the global NH_3_ trade-related health-effect network characteristics.

1. *Degree and degree distribution*: in the health-effect network, definitions of out-degree and in-degree are analogous to those in trade network^46–48^. Out-degree is the number of economies to which a given economy bears health loss for exporting goods, and in-degree is the number of economies that a given economy is transferring health loss to by importing goods from them. The two indicators measure the extensive margin of the economy involved in international trade and are defined as
  9 $${k}_{i}^{{{{{\mathrm{out}}}}}}=\mathop{\sum }\limits_{j\,=\,1(i\,\ne\, j)}^{n}{a}_{ij},\,{k}_{i}^{{{{{\mathrm{in}}}}}}=\mathop{\sum }\limits_{j\,=\,1(i\,\ne\, j)}^{n}{a}_{ji}$$
  where ${a}_{ij}$ is a dummy variable indicating whether health effects are flowing from economy $i$ to economy $j$, $n$ is the total number of economies (181 in the health-effect network), ${k}_{i}^{{{{{\mathrm{out}}}}}}$ and ${k}_{i}^{{{{{\mathrm{in}}}}}}$ represent the out-degree and in-degree, respectively.
  To further analyze the heterogeneity among the 181 economies, we calculate the probability distribution of degree $k$ as $p(k)={n}_{k}/n$, where ${n}_{k}$ is the number of economies that have the same degree $k$. The network can be characterized as a scale-free network if its degree distribution is well fitted by a power-law distribution, i.e., $p(k)\propto {k}^{-\lambda }$. A scale-free network implies the coexistence of a large number of nodes in the periphery that are loosely connected with others and a very few hub nodes that play central roles in connecting other nodes.
  If we consider the weighted health-effect network, links connecting any two economies are not regarded as binary indicators but weighted in proportion to the health-effect flows between them. Just analogous to out-degree and in-degree, out-strength and in-strength can be obtained by replacing ${a}_{ij}$ with ${q}_{ij}$, which indicates the volume of health effects transferred from $i$ to $j$:
  10 $${s}_{i}^{{{{{\mathrm{out}}}}}}= \mathop{\sum }\limits_{j\,=\,1(i\,\ne\, j)}^{n}{q}_{ij},\,{s}_{i}^{{{{{\mathrm{in}}}}}}=\mathop{\sum }\limits_{j\,=\,1(i\,\ne\, j)}^{n}{q}_{ji}$$
2. *Betweenness centrality*: betweenness centrality characterizes the connectivity and intermediality of a network and reflects the importance of a given node as the role of bridging other nodes by calculating the number of shortest paths that go through it:
  11 $${b}_{k}=\mathop{\sum }\limits_{i\,=\,1}^{n}\mathop{\sum }\limits_{j\,=\,1}^{n}{\sigma }_{ij}(k)/{\sigma }_{ij}$$
  where ${\sigma }_{ij}$ is the number of shortest paths between economy $i$ and economy $j$, ${\sigma }_{ij}(k)$ is the number of shortest paths between $i$ and $j$ that pass through economy $k$, ${b}_{k}$ is the betweenness centrality of economy $k$. This measure indicates if economy $k$ is on the shortest path between $i$ and $j$, then it counts towards the betweenness centrality of economy $k$. In the health-effect network, an economy with high betweenness centrality implies its crucial bridging roles in transferring or receiving health effects.
  For the weighted health-effect network, the path length from economy $i$ to economy $j$ is defined by the number of bilateral flows of health effects ${q}_{ij}$, with which we likewise obtain the weighted betweenness centrality.
3. *Eigenvector centrality*: another prevalent centrality measure is the eigenvector centrality, which evaluates the importance of a node based on its neighboring nodes. The intuition behind this is that a node should have high centrality if it is connected with many other nodes that also have high eigenvector centrality. Eigenvector centrality is defined as:
  12 $${{{{{{\bf{v}}}}}}}_{{{{{{\bf{i}}}}}}}={\lambda }^{-1}\mathop{\sum }\limits_{j\,=\,1}^{n}{a}_{ij}{{{{{{\bf{v}}}}}}}_{{{{{{\bf{j}}}}}}}$$
  where $\lambda$ and ${{{{{{\bf{v}}}}}}}_{{{{{{\bf{j}}}}}}}$ are the largest eigenvalue and the corresponding eigenvector.
4. *The weighted average of nearest-neighbor degree*: a similar hybrid network is a network in which nodes tend to be connected with other nodes of similar degree. To assess this tendency, we calculate the weighted neighboring degree of node $i$:
  13 $${\omega }_{i}=\mathop{\sum }\limits_{j\,=\,1}^{v(i)}({q}_{ij}+{q}_{ji})({k}_{j}^{{{{{\mathrm{out}}}}}}+{k}_{j}^{{{{{\mathrm{in}}}}}})/({s}_{i}^{{{{{\mathrm{out}}}}}}+{s}_{i}^{{{{{\mathrm{in}}}}}})$$
  where $v(i)$ is the number of the neighboring nodes of economy $i$.
  Given that the degree of economy $i$ is $k$, the average neighboring degree of all the nodes with degree $k$ is defined as:
  14 $$\omega (k)=\mathop{\sum }\limits_{i\in \{j|{k}_{j}^{{{{{\mathrm{out}}}}}}+{k}_{j}^{{{{{\mathrm{in}}}}}}=k\}}^{{n}_{k}}{\omega }_{i}/{n}_{k}$$
  The network is a similar hybrid network if and only if $\omega (k)$ is monotonically increasing in $k$.
5. *Community detection*: in order to better visualize the health-effect network, it is useful to partition the complex network, which consists of 181 nodes and more than 30,000 edges, into several submodules or communities, within which the nodes are densely linked but sparsely connected with the nodes in other communities. We apply the modularity maximization method introduced by Girvan and Newman^29^ to find the community partition of the health-effect network. The modularity of partition compares the compactness of the links inside communities with the links between communities. A higher value of modularity suggests better quality of community partition. The modularity $Q$ in our network is defined by:
  15 $$Q=\frac{1}{2m}\mathop{\sum }\limits_{i\,=\,1}^{n}\mathop{\sum }\limits_{j\,=\,1}^{n}\left[{w}_{ij}-\frac{{p}_{i}{p}_{j}}{2m}\right]\delta ({c}_{i},{c}_{j})$$
  where ${w}_{ij}={q}_{ij}+{q}_{ji}$ is the amount of health effects connection between economy $i$ and economy $j$, ${p}_{i}=\mathop{\sum }\nolimits_{j\,=\,1}^{n}{w}_{ij}$ is the sum of health effects attached to economy $i$, ${c}_{i}$ is the community to which economy $i$ is assigned, $\delta ({c}_{i},{c}_{j})$ is an indicator function which equals to 1 if ${c}_{i}={c}_{j}$ and 0 otherwise, and $m=\mathop{\sum }\nolimits_{i\,=\,1}^{n}\mathop{\sum }\nolimits_{j\,=\,1}^{n}{w}_{ij}/2$. We use this algorithm to implement the method and extract community structures of the network.

### Chemical transport model

The GEOS-Chem is a global three-dimensional CTM of the atmospheric compositions (version 12.0.0, http://www.geos-chem.org), and includes detailed ozone–NO_*x*_–VOC–aerosol chemistry^49^. The model was run at a horizontal resolution of 2° latitude by 2.5° longitude driven by the NASA Modern-Era Retrospective Analysis for Research and Applications, Version 2 (MERRA-2) meteorological fields. In GEOS-Chem simulations, NH_3_ emissions from anthropogenic sources were from EDGAR v4.3.2 for 2012, and emissions from soil, vegetation, and the oceans were from the Global Emissions Inventory Activity inventory^50^. Other global anthropogenic emissions of NO_*x*_, SO_2_, CO, black carbon (BC), and organic carbon (OC) from EDGAR v4.3.2 and speciated volatile organic compounds emissions from the RETRO, overwritten by the default regional emissions, were adopted. Other natural emissions follow the configuration of Li et al.^51^.

A baseline simulation was conducted driven by global anthropogenic and natural emissions described above. To quantify the impacts of export-related agricultural NH_3_ emissions on particulate air pollution, sensitivity simulations with deducted NH_3_ emissions embodied in international trade for 181 countries were also performed. For each country, the trade-related fraction of NH_3_ emissions is assumed uniform, and this method has been applied widely in the previous studies^21,22,52^. The fractional contributions of export-related NH_3_ emissions to PM_2.5_ were determined on a 2° latitude by 2.5° longitude grid, due to model resolution. Then, these spatially varying fractions were multiplied by the 0.1° × 0.1° global PM_2.5_ concentrations from GBD 2013^28^ to get estimated PM_2.5_ concentrations induced by export-related NH_3_ emissions. All the simulations were conducted from January to December 2012 after a 6-month model initialization (July–December 2011).

GEOS-Chem aerosol simulations have been extensively evaluated using ground-based measurements worldwide^2,11,22,53–57^, including the USA, Europe, China, and India. These previous studies have shown that the GEOS-Chem model can reasonably the response of PM_2.5_ formation to emission changes as well as the observed concentrations of PM_2.5_ components. For example, Zhang et al.^22^ reported that major PM_2.5_ components simulated by GEOS-Chem have an *R*^2^ of 0.52~0.78 when compared with those observed values over the US, Europe, and East Asia, while it tends to underestimate (overestimate) BC (nitrate). The high bias of nitrate and low bias of BC are the common issues in the GEOS-Chem model^11,58^. It means that further improvement of the model’s capability in capturing the dynamics of the sulfate–nitrate–ammonium aerosol systems is needed. Here we used GBD-based PM_2.5_ concentrations in 2012 to validate the model-simulated PM_2.5_ concentrations (Supplementary Fig. 5). The simulated and GBD-based PM_2.5_ concentrations (with a resolution of 0.1° × 0.1°) have a correlation coefficient of 0.6 and normalized mean bias of −0.5%. These two datasets compare reasonably well for most regions, including high values of over 80 μg m^−3^ over eastern China and northern India, higher values in the eastern US than the western US, and high values of about 35 μg m^−3^ over some European areas.

### Assessment of health impacts

Methods of the GBD study^28^ are followed to estimate the premature deaths from ambient PM_2.5_ exposure. Here the impacts due to the four leading causes of death: ischemic heart disease, chronic obstructive pulmonary disease, cerebrovascular disease, and lung cancer are considered. In 2012, these four major diseases together accounted for 18.4 million deaths (∼35% of all-cause mortality). We estimate 3.54 million premature deaths attributable to PM_2.5_ in 2012, which agrees well with some previous studies such as the GBD^59^ (3.44 million deaths) and Zhang et al.^22^ (3.45 million deaths).

We applied IER functions developed by Burnett et al.^59^, which incorporated data from cohort studies of ambient PM_2.5_ pollution, household air pollution, and active and passive tobacco smoke, to fit the concentration–response relationship throughout the full distribution of ambient PM_2.5_ concentrations. Thus, high PM_2.5_ concentrations similar to those observed in China and India can be also accounted for. For each disease, the relative risk for mortality estimations for the all-age group was calculated as the following equation:

16 $${{{{{\mathrm{RR}}}}}}(C) 	=1+\alpha \{1-\exp [-\gamma {(C-{C}_{0})}^{\delta }]\}\,{{{{{\mathrm{for}}}}}}\,C \, > \, {C}_{0}\\ \,{{{{{\mathrm{RR}}}}}} 	=1\,{{{{{\mathrm{for}}}}}}\,C\le {C}_{0}$$

where *C* represents the annual mean PM_2.5_ concentration (on a 0.1° × 0.1° grid) in 2012, which was exponentially extrapolated from data for 2010 based on the GBD study by Brauer et al.^28^. The calibrated GBD PM_2.5_ data were estimated by a combination of satellite-based estimates, chemical transport model simulations, and ground measurements; *C*_0_ is the counterfactual concentration, representing a theoretical minimum-risk concentration above which there is evidence indicating health benefits of PM_2.5_ exposure reductions (range: 5.8 − 8.8 μg m^−3^); and $\alpha$,$\,\gamma$, and $\delta$ are parameters used to determine the overall shape of the concentration–response relationship, which are obtained from Burnett et al.^59^. We reported averaged mortality results using 1000 sets of coefficients and exposure–response functions based on Monte Carlo simulations.

The grid-based (0.1° × 0.1°) premature deaths attributed to ambient PM_2.5_ exposures were then estimated:

17 $${{{{{\mathrm{Mort}}}}}}={y}_{0}\,\times \,{{{{{\mathrm{pop}}}}}}\,\times \,(1-1/{{{{{\mathrm{RR}}}}}})$$

where ${y}_{0}$ is the Country-level baseline mortality for each disease for the all-age group from the Institute for Health Metrics and Evaluation (http://ghdx.healthdata.org/ihme_data) and pop is the population obtained from the Gridded Population of the World, version 3 at a resolution of 2.5min   × 2.5 min, which was further aggregated to the same resolution of 0.1° × 0.1°. The estimated 2012 population is linearly extrapolated from the 2010 and 2011 values.

Here, we estimated the mortality contribution from export-related agricultural NH_3_ emissions based on an assumption that the contribution of one source to the disease burden of PM_2.5_ is directly proportional to its share of PM_2.5_ concentration. The more recent GBD research, GBD MAPS, has demonstrated the scientific basis of such an assumption, which was adopted by early studies, such as another GBD study^60^ and Zhang et al.^22^. For a given country, the premature deaths from its export-related NH_3_ emissions can be calculated by multiplying its fractional contribution of export-related NH_3_ emissions to PM_2.5_ concentration by the total PM_2.5_ concentration-related mortalities for each 0.1° × 0.1° grid cell. The fractional contribution of export-related NH_3_ emissions to PM_2.5_ was estimated by the GEOS-Chem simulations.

### Trade structure adjustment scenarios

Scenario analysis is conducted through import substitution and export transfer within the countries in each community.

Suppose that country *i* imports ${{{{{\mathrm{I}}}}}}{{{{{\mathrm{M}}}}}}_{ij}$ agricultural products from country *j*, and the NH_3_ emission intensities of the two countries are ${{{{{\mathrm{E}}}}}}{{{{{\mathrm{I}}}}}}_{i}$ and ${{{{{\mathrm{E}}}}}}{{{{{\mathrm{I}}}}}}_{j}$. If ${{{{{\mathrm{E}}}}}}{{{{{\mathrm{I}}}}}}_{i} \, < \, {{{{{\mathrm{E}}}}}}{{{{{\mathrm{I}}}}}}_{j}$, emission reduction can be achieved by country *i* to substitute the import ${{{{{\mathrm{I}}}}}}{{{{{\mathrm{M}}}}}}_{ij}$ with its own production. Denote the amount substituted as ${{{{{\mathrm{S}}}}}}{{{{{\mathrm{T}}}}}}_{ij}$. For any country *i*, the objective function of import substitution is to maximize the emission reduction, i.e., $\mathop{\sum }\nolimits_{j=1,j\ne i}^{n}({{{{{\mathrm{E}}}}}}{{{{{\mathrm{I}}}}}}_{j}-{{{{{\mathrm{E}}}}}}{{{{{\mathrm{I}}}}}}_{i})\times {{{{{\mathrm{S}}}}}}{{{{{\mathrm{T}}}}}}_{ij}$. Nevertheless, complete substitution is unrealistic due to constraints in natural resources. Countries are unable to expand their agricultural production beyond their total capacity. We impose this constraint with their potential capacity in agricultural production (denoted as ${{{{{\mathrm{A}}}}}}{{{{{\mathrm{C}}}}}}_{i}$), measured by the area of arable land (in hectare) multiplied by the value of agricultural products per hectare. Furthermore, farm goods produced in different countries are not perfectly substitutable either. To increase the reliability of the counterfactual analysis, we limit trade substitution within countries with annual average temperature (denoted as AVT) and precipitation (denoted as PCP) at similar conditions ($\pm$5 °C for temperature and $\pm$500 mm for precipitation). Therefore, for country *i*, the objective function of import substitution is:

18 $$\mathop{\max }\limits_{\{{{{{{\mathrm{S}}}}}}{{{{{\mathrm{T}}}}}}_{ij}\}}\mathop{\sum }\limits_{j=1,j\ne i}^{n}({{{{{\mathrm{E}}}}}}{{{{{\mathrm{I}}}}}}_{j}-{{{{{\mathrm{E}}}}}}{{{{{\mathrm{I}}}}}}_{i})\times {{{{{\mathrm{S}}}}}}{{{{{\mathrm{T}}}}}}_{ij}$$

$${{{{{\mathrm{s.t.}}}}}}\mathop{\sum }\limits_{j=1,j\ne i}^{n}{{{{{\mathrm{S}}}}}}{{{{{\mathrm{T}}}}}}_{ij}\le {{{{{\mathrm{A}}}}}}{{{{{\mathrm{C}}}}}}_{i}$$

$$0\le {{{{{\mathrm{S}}}}}}{{{{{\mathrm{T}}}}}}_{ij}\le {{{{{\mathrm{I}}}}}}{{{{{\mathrm{M}}}}}}_{ij}$$

$$-5\le {{{{{\mathrm{AV}}}}}}{{{{{\mathrm{T}}}}}}_{i}-{{{{{\mathrm{AV}}}}}}{{{{{\mathrm{T}}}}}}_{j}\le 5$$

$$-500\le {{{{{\mathrm{PC}}}}}}{{{{{\mathrm{P}}}}}}_{i}-{{{{{\mathrm{PC}}}}}}{{{{{\mathrm{P}}}}}}_{j}\le 500$$

The import substitution involves two parties, i.e. the exporter and importer. More substantial emission reduction will be achieved if allowing the production of those exported products to be transferred to a third country with lower emission intensity than the original exporter and importer. The scenario of export transfer we consider is to minimize the total NH_3_ emissions in agricultural trade for each community. This is equivalent to taking total exports as given, reorganizing the production structure of exported goods within countries of the same community. Therefore, the objective function for export transfer is:

19 $$\mathop{\max }\limits_{\{{{{{{\mathrm{E}}}}}}{{{{{\mathrm{X}}}}}}_{i}^{{{{{\mathrm{AF}}}}}}\}}\mathop{\sum }\limits_{i\,=\,1}^{n}{{{{{\mathrm{E}}}}}}{{{{{\mathrm{X}}}}}}_{i}^{{{{{\mathrm{AF}}}}}}\times {{{{{\mathrm{E}}}}}}{{{{{\mathrm{I}}}}}}_{i}$$

$${{{{{\mathrm{s.t.}}}}}}\mathop{\sum }\limits_{i\,=\,1}^{n}{{{{{\mathrm{E}}}}}}{{{{{\mathrm{X}}}}}}_{i}^{0}=\mathop{\sum }\limits_{i\,=\,1}^{n}{{{{{\mathrm{E}}}}}}{{{{{\mathrm{X}}}}}}_{i}^{{{{{\mathrm{AF}}}}}}$$

$${{{{{\mathrm{E}}}}}}{{{{{\mathrm{X}}}}}}_{i}^{{{{{\mathrm{AF}}}}}}\le {{{{{\mathrm{A}}}}}}{{{{{\mathrm{C}}}}}}_{i}+{{{{{\mathrm{E}}}}}}{{{{{\mathrm{X}}}}}}_{i}^{0}$$

$${{{{{\mathrm{E}}}}}}{{{{{\mathrm{X}}}}}}_{i}^{0}\le \mathop{\sum }\limits_{j\,=\,1}^{n}{I}_{ij}\times {{{{{\mathrm{E}}}}}}{{{{{\mathrm{X}}}}}}_{j}^{{{{{\mathrm{AF}}}}}}$$

where ${{{{{\mathrm{E}}}}}}{{{{{\mathrm{X}}}}}}_{i}^{0}$ is the original export of country *i* before the adjustment of export transfer, ${{{{{\mathrm{E}}}}}}{{{{{\mathrm{X}}}}}}_{i}^{{{{{\mathrm{AF}}}}}}$ is the new export of country *i* after the adjustment, ${I}_{ij}$ is a dummy variable which equals 1 if and only if $-5\le {{{{{\mathrm{AV}}}}}}{{{{{\mathrm{T}}}}}}_{i}-{{{{{\mathrm{AV}}}}}}{{{{{\mathrm{T}}}}}}_{j}\le 5$ and $-500\le {{{{{\mathrm{PC}}}}}}{{{{{\mathrm{P}}}}}}_{i}-{{{{{\mathrm{PC}}}}}}{{{{{\mathrm{P}}}}}}_{j}\le 500$. This first constraint keeps the total exports unchanged after the adjustment. The second constraint ensures the adjusted export of country *i* does not exceed its total production capacity, measured by the sum of potential capacity and original exports. The last constraint guarantees that the original export of country *i* is transferred to countries with similar annual temperature and precipitation.

We solve the above linear optimization problems to find the maximized emission reduction under export transfer and import substitution. The area of agricultural land and arable land is obtained from FAO (http://www.fao.org/faostat/en/#data/RL). Average annual temperature and rainfall are measured by the country-level averages from 1990 to 2012, which are available from the Climatic Research Unit of University of East Anglia (http://www.cru.uea.ac.uk/).

### Technological advancement scenarios

We develop several NH_3_ emission control scenarios from the production side (reducing N fertilizer overuse, deep placement of fertilizers, enhanced-efficiency fertilizers, and improved animal manure storage and disposal) and consumption side (reducing food loss and waste, and replacing beef consumption with soy consumption).

1. *Estimating the potential of reducing N fertilizer overuse*: N fertilizers are applied in unnecessarily high amounts in much of Asia (especially China), India, USA, and Europe. Overuse of N is much more severe in vegetables and fruits production, e.g., in China. Management of N during fruits and vegetable production has a much larger potential to improve compared to management during grain crop production. Mueller et al.^61^ estimated that nitrogen-fertilizer application on maize, wheat, and rice could decrease globally by 28% without impacting current yields. Considering the share of crop grain crop N in total N fertilizer use, we conservatively estimate that globally total N fertilizer use can be reduced by 14% to remove oversupply of nutrients and associated Nr losses. It is also noted that N fertilizer might not be reduced if there is not a regime to replace existing inorganic fertilizer applications.
2. *Estimating the potential of fertilizer deep placement and enhanced-efficiency fertilizers in grain crops*: deep placement of N fertilizers can make N less susceptible to NH_3_ volatilization and more available to grain crops. A global meta-analysis found 55% NH_3_ emission reduction achieved through deep placement^62^. A meta-analysis for China found 35% NH_3_ emission reduction achieved for wheat and rice systems and 70% for maize. We conservatively estimate that 55% of grain production-related NH_3_ emissions can be reduced through deep placement. We obtain the contribution of grain production to total crop NH_3_ from Paulot et al.^63^. Urease inhibitors reduce the hydrolysis rate of urea fertilizers thus reducing NH_3_ emission rates by 40–70% depending on crop types and N application rates^64^. A global meta-analysis of field experiments reported 54% NH_3_ emission reduction according to 198 observations^62^. A meta-analysis for China found 35% NH_3_ emission reduction achieved for wheat and rice systems and 70% for maize^65^. A field research in Germany estimated 70% NH_3_ emission reduction^66^. We conservatively estimate that urease inhibitors will reduce NH_3_ emissions from grain crops by 54%.
3. *Estimating the mitigation potential from livestock production*: A global meta-analysis found the highest mitigation potential in dietary additive (35–54%), urease inhibitor (24–69%), manure acidification (89–95%), and deep manure placement (94–99%). Manure storage management could also significantly reduce NH_3_ emission by 70–82%^67^. These mitigation measures should be taken simultaneously to effectively reduce NH_3_ emissions from livestock sectors. Despite great technological potential to reduce NH_3_ emissions, currently, manure around the world has been poorly managed^68^. For example, two-thirds of manure N produced in China are released as air pollutants^69^. We thus conservatively estimate that moderate improvements in manure management can reduce NH_3_ emissions by 35% and drastic improvements can reduce 70%.
4. *Estimating the potential of eliminating food loss and waste*: globally around 1/3 of food produced are discarded during the food supply chain, food retail and consumption processes^70^. Reducing food waste and loss thus provides the opportunity for reducing agricultural emissions, especially in developed regions such as Europe and North America which already have relatively effective production management. We estimate the NH_3_ mitigation potential of eliminating food waste and loss for each country using the following equation:
  20 $${{Reductio}}{{n}}_{{{NH}}_{3}} 	={{Reductio}}{{n}}_{{{cro}}{{p}}_{{{NH}}_{3}}}+{{Reductio}}{{n}}_{{{livestoc}}{{k}}_{{{NH}}_{3}}}\\ 	={{Baselin}}{{e}}_{{{cro}}{{p}}_{{{NH}}_{3}}}\times {{Wast}}{{e}}_{{{LossRati}}{{o}}_{{crop}}}\\ 	\kern1pc+\, {{Baselin}}{{e}}_{{{livestoc}}{{k}}_{{{NH}}_{3}}}\times {{Waste}}\_{{Loss}}\_{{Rati}}{{o}}_{{meat}}$$
  where ${{{{{\mathrm{Baselin}}}}}}{{{{{\mathrm{e}}}}}}_{{{{{{\mathrm{cro}}}}}}{{{{{\mathrm{p}}}}}}_{{{{{{\mathrm{NH}}}}}}_{3}}}$ and ${{{{{\mathrm{Baselin}}}}}}{{{{{\mathrm{e}}}}}}_{{{{{{\mathrm{livestoc}}}}}}{{{{{\mathrm{k}}}}}}_{{{{{{\mathrm{NH}}}}}}_3}}$ are national total NH_3_ emissions from N fertilizer application and livestock manure handling, respectively, for the year 2012 from the EDGAR inventory, ${{{{{\mathrm{Waste}}}}}}\_{{{{{\mathrm{Loss}}}}}}\_{{{{{\mathrm{Rati}}}}}}{{{{{\mathrm{o}}}}}}_{{{{{\mathrm{crop}}}}}}$ and ${{{{{\mathrm{Waste}}}}}}\_{{{{{\mathrm{Loss}}}}}}\_{{{{{\mathrm{Rati}}}}}}{{{{{\mathrm{o}}}}}}_{{{{{\mathrm{meat}}}}}}$ are the ratios of food loss and waste of cereal crops and animal meat products in this country. ${{{{{\mathrm{Waste}}}}}}\_{{{{{\mathrm{Loss}}}}}}\_{{{{{\mathrm{Ratio}}}}}}$ are estimated to include food loss and waste during agricultural production, postharvest handling and storage, processing and packaging, distribution and supermarket retail, and food consumption for each major region provided by Gustavsson et al.^71^.
5. *Estimating the potential of dietary shifts*: beef, compared to other animal meat products, have much heavier nitrogen and water use footprints and greenhouse gas emissions^72^. NH_3_ emissions from beef manure alone contributes to 30% of agricultural NH_3_ emissions globally^63^. Replacing beef protein with other animal meat products or plant-based soybean protein can help reduce NH_3_ emissions. Here we consider the dietary change strategy of reducing beef consumption by 20% and 50%, replacing that beef protein with soybean protein. The additional NH_3_ emissions brought by increased soybean cultivation are negligible. This is because the reduced animal feed production (mostly soy) should more than enough cover the increased soybean consumption, given that protein in animal feed does not end 100% in beef products. In addition, soybean is a N-fixing crop and requires moderate N fertilizer application and emits little NH_3_ emissions. We obtain the contribution of beef production to NH_3_ emissions from animal production in the US, Europe, China and the world from Paulot et al.^63^. Then on top of EDGAR NH_3_ emission inventory, we impose a 20% and a 50% decrease of beef NH_3_ emissions by country.
6. *The MTFR scenario from GAINS model*: we also applied the MTFR scenario by 2050 calculated from the GAINS (Greenhouse gas-Air pollution Interactions and Synergies) model (freely online from the website: https://gains.iiasa.ac.at/models/gains_models3.html), which is developed by the International Institute for Applied Systems Analysis (IIASA)^36^. The GAINS model has been widely applied for assessing strategies of ammonia emission abatement^73,74^. The MTFR scenario in GAINS model assumes implementation of best available measures ignoring political or economic constraints but considering technical applicability that might vary regionally.

### Uncertainty

Export-related NH_3_ emissions link local producers to global consumers along the entire supply chain. Based on multimodels, the uncertainty in this study mainly lies in the emissions inventory, economic data that includes the national accounts and interregional trade, and atmospheric transport model, and atmospheric model. Previous studies quantifying the uncertainty of national consumption-based carbon emissions (including imports and excluding the exports) are in the range 5–15%^75^ and 2–16%^76^. The comparable uncertainty range of production- and consumption-based accounts indicate that a major source of uncertainty of MRIO is mainly the emission inventory rather than the economic and trade data^77^. According to Crippa et al.^27^, the uncertainty of EDGAR NH_3_ emissions is within a factor of 2–3 for global major regions. This is mainly due to the uncertainty of adopted emission factors. Van Damme et al.^3^ has shown that the EDGAR inventory is able to capture NH_3_ emissions in the large source regions while it fails to capture strong point sources. Other observation-based constraints of NH_3_ emissions also have confirmed the validation of EDGAR NH_3_ inventory in China, USA, and Europe^63,78^. Future studies to further improve the accuracy of NH_3_ emissions in global inventories are urgently called for.

### Reporting summary

Further information on research design is available in the Nature Research Reporting Summary linked to this article.

## Supplementary information

Supplementary Information

Description of Additional Supplementary Files

Supplementary Data 1

Supplementary Data 2

Supplementary Data 3

Supplementary Data 4

Supplementary Data 5

Supplementary Data 6

Reporting Summary

## Data Availability

The EDGAR v4.3.2 emission database is from Joint Research Centre, European Commission (https://data.jrc.ec.europa.eu/dataset/jrc-edgar-v432-ap-gridmaps). The global MRIO tables are from https://worldmrio.com/countrywise/. The Eora database is freely accessible from https://www.worldmrio.com/eora/. The United Nations Comtrade Database is available from https://comtrade.un.org/. The population and GDP data are available from the statistical database of the World Bank (https://data.worldbank.org). Country-level baseline mortality for the all-age group from the Institute for Health Metrics and Evaluation (http://ghdx.healthdata.org/ihme_data). The area of agricultural land and arable land is obtained from FAO (http://www.fao.org/faostat/en/#data/RL). Average annual temperature and precipitation data are available from the Climatic Research Unit of University of East Anglia (http://www.cru.uea.ac.uk/). The GAINS model is from the International Institute for Applied Systems Analysis (IIASA) (https://gains.iiasa.ac.at/models/gains_models3.htm).

## Code availability

The GEOS-Chem model code is open-source 10.5281/zenodo.1343547. Codes for reproducing the results in this study are available from https://github.com/KeLi218/NH3_emissions/ and also available from corresponding authors on reasonable request.
