# Publisher Correction: Mitigation potential of global ammonia emissions and related health impacts in the trade network

**DOI:** 10.1038/s41467-021-27476-1

**Published:** 2021-11-30

**Authors:** Rong Ma, Ke Li, Yixin Guo, Bo Zhang, Xueli Zhao, Soeren Linder, ChengHe Guan, Guoqian Chen, Yujie Gan, Jing Meng

**Affiliations:** 1 School of Economics and Management, Beihang University, Beijing, China; 2 Harvard–NUIST Joint Laboratory for Air Quality and Climate, Jiangsu Key Laboratory of Atmospheric Environment Monitoring and Pollution Control, Collaborative Innovation Center of Atmospheric Environment and Equipment Technology, School of Environmental Science and Engineering, Nanjing University of Information Science and Technology, Nanjing, China; 3 John A. Paulson School of Engineering and Applied Sciences, Harvard University, Cambridge, MA USA; 4 Princeton School of Public and International Affairs, Princeton University, Princeton, NJ USA; 5 School of Management, China University of Mining and Technology (Beijing), Beijing, China; 6 Joint Research Centre, Food Security Group, European Commissions, Ispra, Italy; 7 Arts and Science, New York University Shanghai, Shanghai, China; 8 Laboratory of Systems Ecology and Sustainability Science, College of Engineering, Peking University, Beijing, China; 9 School of Government, The Leo KoGuan Building, Peking University, 100871 Beijing, China; 10 The Bartlett School of Sustainable Construction, University of College London, London, WC1E 7HB UK; 11 Present Address: Laboratory for Climate and Ocean–Atmosphere Studies, Department of Atmospheric and Oceanic Sciences, School of Physics, Peking University, Beijing, China

**Keywords:** Atmospheric chemistry, Environmental impact, Environmental economics, Sustainability

Correction to: *Nature Communications* 10.1038/s41467-021-25854-3, published online 5 November 2021.

In this article the affiliation details for Guoqian Chen were incorrectly given as ‘Laboratory of Systems Ecology and Sustainability Science, College of EngineeS-Chem model code is open-souring, Peking University, Beijing, China' but should have been ‘Laboratory of Systems Ecology and Sustainability Science, College of Engineering, Peking University, Beijing, China’. The original article has been corrected.

