# Supplementary material for: Mitigation potential of global ammonia emissions and related health impacts in the trade network
Source: Nat Commun. 2021 Nov 5;12:6308. doi: 10.1038/s41467-021-25854-3 (PMC8571346; doi:10.1038/s41467-021-25854-3)
Supplement: Supplementary file 1 — Supplementary Information [file 41467_2021_25854_MOESM1_ESM.pdf]

Supplementary Information for

Mitigation potential of global ammonia emissions and related health impacts in the trade network

Rong Ma[1#], Ke Li[2,3#], Yixin Guo[4,11], Bo Zhang[5*], Xueli Zhao[5], Soeren Linder[6], ChengHe Guan[7], Guoqian Chen[8], Yujie Gan[9] and Jing Meng[10*]

[1]School of Economics and Management, Beihang University, Beijing, China

[2]Harvard–NUIST Joint Laboratory for Air Quality and Climate, Jiangsu Key Laboratory of Atmospheric Environment Monitoring and Pollution Control, Collaborative Innovation Center of Atmospheric Environment and Equipment Technology, School of Environmental Science and Engineering, Nanjing University of Information Science and Technology, Nanjing, China

[3]John A. Paulson School of Engineering and Applied Sciences, Harvard University, Cambridge, MA, United States

[4]Princeton School of Public and International Affairs, Princeton University, Princeton, New Jersey, United States

[5]School of Management, China University of Mining & Technology (Beijing), Beijing, China

[6]Joint Research Centre, Food Security Group, European Commissions, Ispra, Italy

[7]Arts and Science, New York University Shanghai, Shanghai, China

[8]Laboratory of Systems Ecology and Sustainability Science, College of Engineering, Peking University, Beijing, China

[9]School of Government, The Leo KoGuan Building, Peking University, Beijing, 100871，China

[10]The Bartlett School of Sustainable Construction, University of College London, London WC1E 7HB, UK

[11]now at Laboratory for Climate and Ocean–Atmosphere Studies, Department of Atmospheric and Oceanic Sciences, School of Physics, Peking University, Beijing, China

[#]These authors contributed equally to this work.

[*]*Corresponding to*: B.Z. (zhangbo@cumtb.edu.cn) and J.M. (jing.j.meng@ucl.ac.uk)

The SI includes:

Supplementary Figures 1–6

Supplementary Tables 1–5

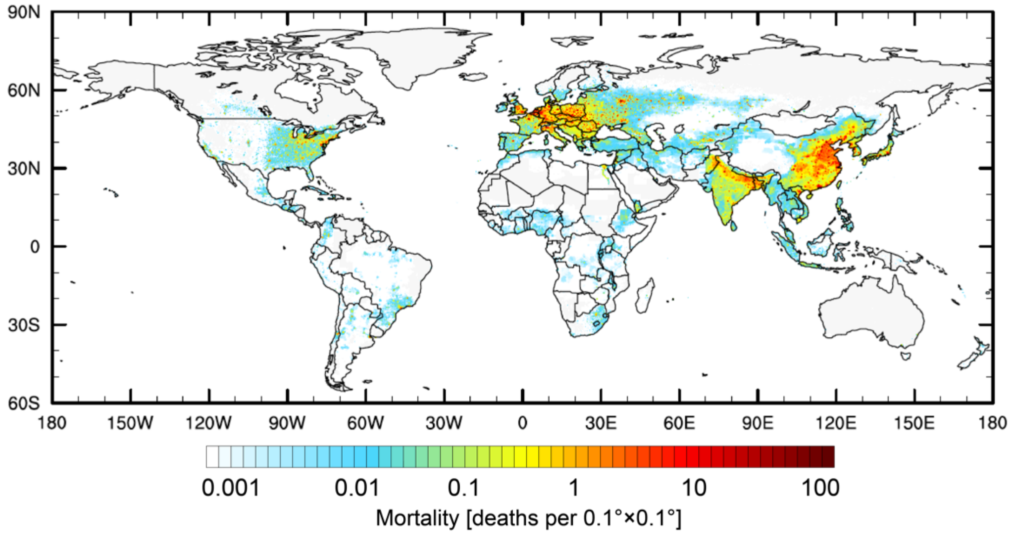

Supplementary Fig. 1 | Total health impacts of export-related NH_3 emissions in 2012. Attributable premature mortality density (deaths per 0.1° × 0.1°) due to total export-related NH_3 emissions from crop cultivation and livestock production. Map was created by using the NCAR Command Language, version 6.4.0 (NCAR, https://doi.org/10.5065/D6WD3XH5).

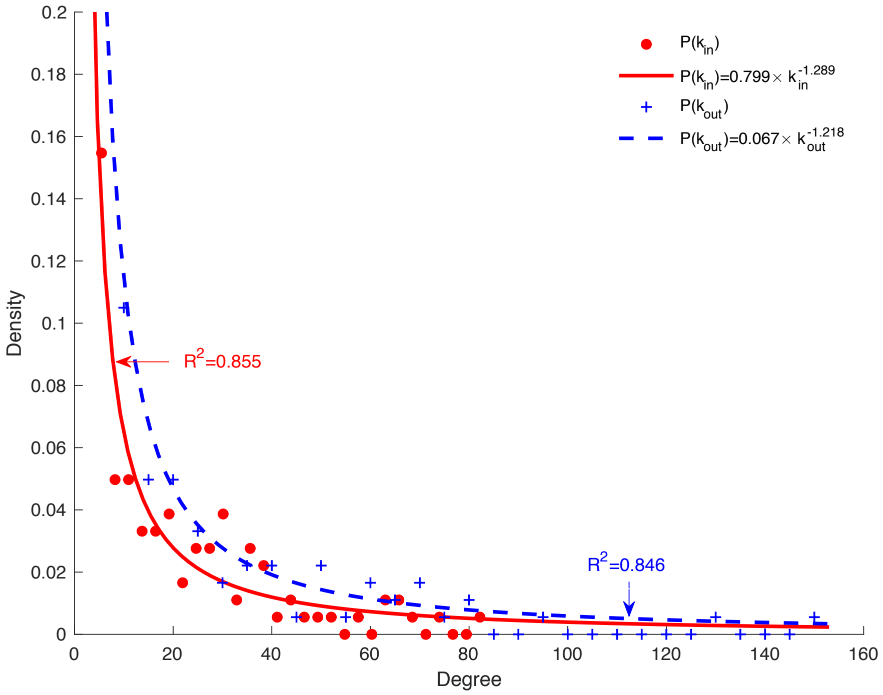

Supplementary Fig. 2 | Degree distribution of the network of NH_3 health effect.

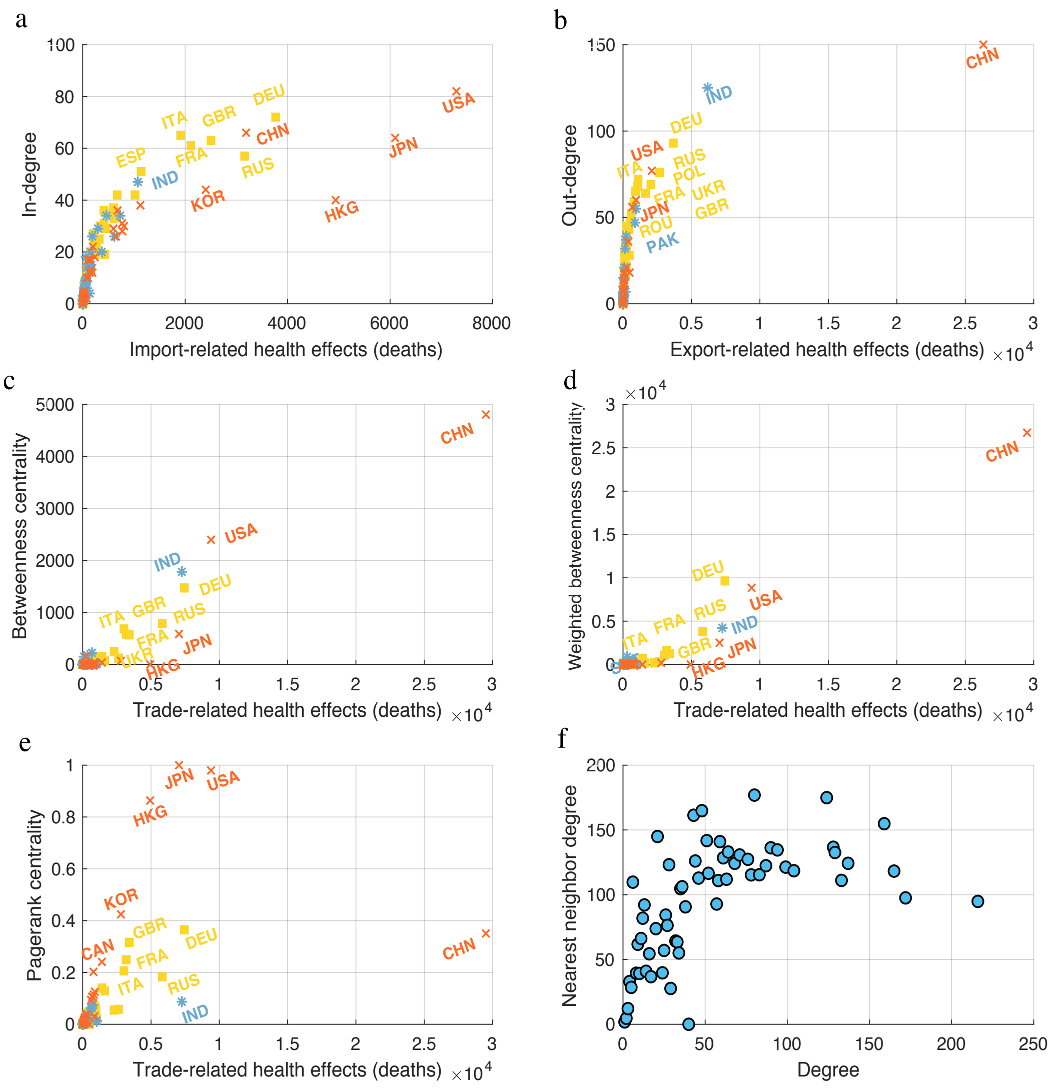

Supplementary Fig. 3 | Health effect network indicators on **a**, out-degree and out-strength, **b**, in-degree and in-strength, **c**, betweenness centrality and strength, **d**, weighted betweenness centrality and strength, **e**, eigenvector centrality and strength, **f**, weighted average of nearest neighbor degree. The three-letter country abbreviations inserted in the plot are detailed in Supplementary Data 6.

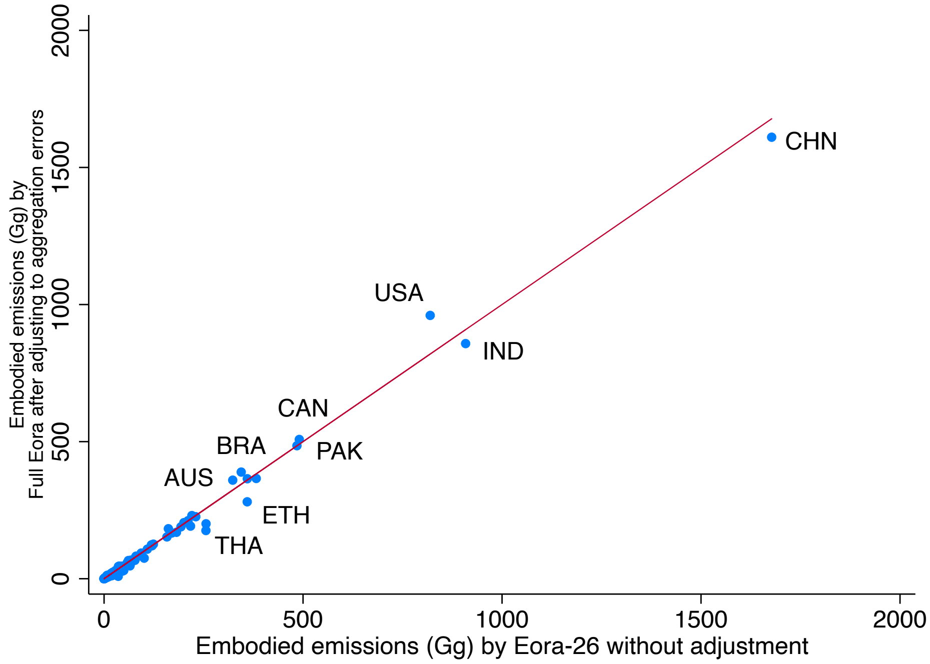

Supplementary Fig. 4 | Embodied agricultural NH_3 emissions (Gg) calculated by the Eora MRIO database without adjust and by the Full Eora database with adjustment to aggregation errors. The three-letter country abbreviations inserted in the plot are detailed in Supplementary Data 6.

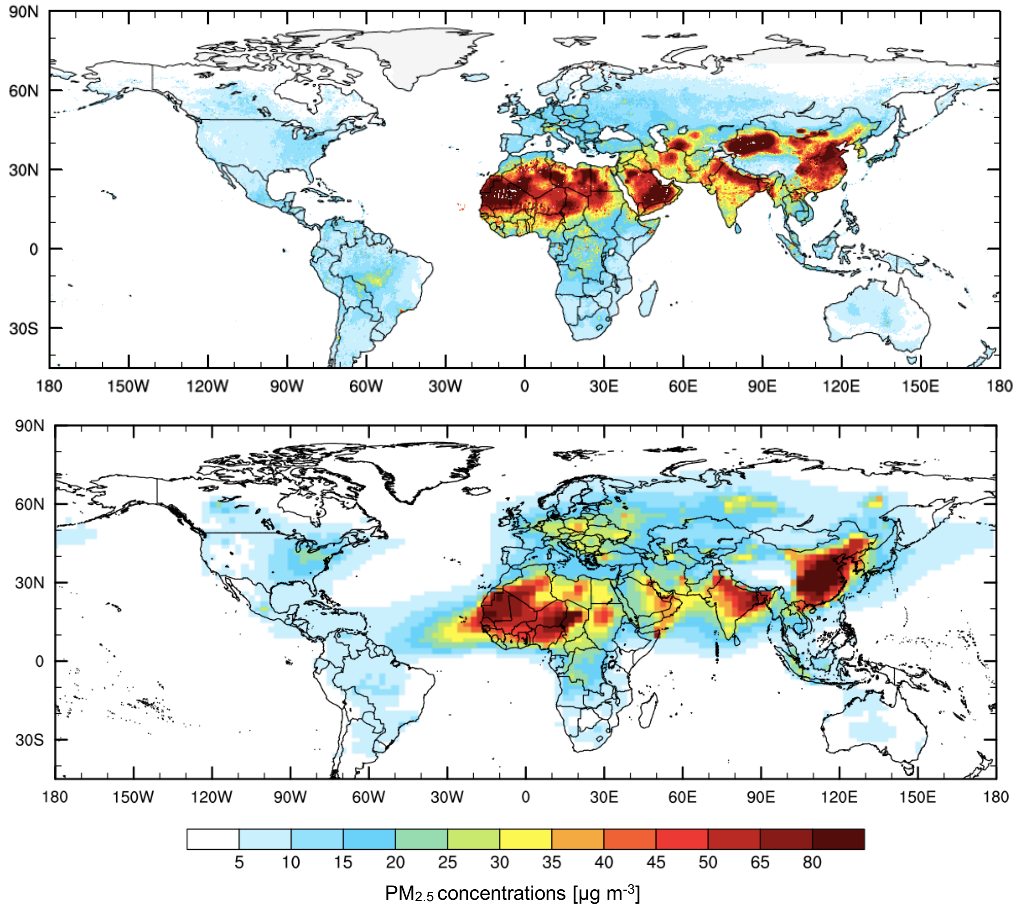

Supplementary Fig. 5 | Annual mean $PM_{2.5}$ concentrations (μg m^{-3}) in 2012 from GBD-based data (*top*) and GEOS-Chem simulation (*bottom*). Maps were created by using the NCAR Command Language, version 6.4.0 (NCAR, https://doi.org/10.5065/D6WD3XH5).

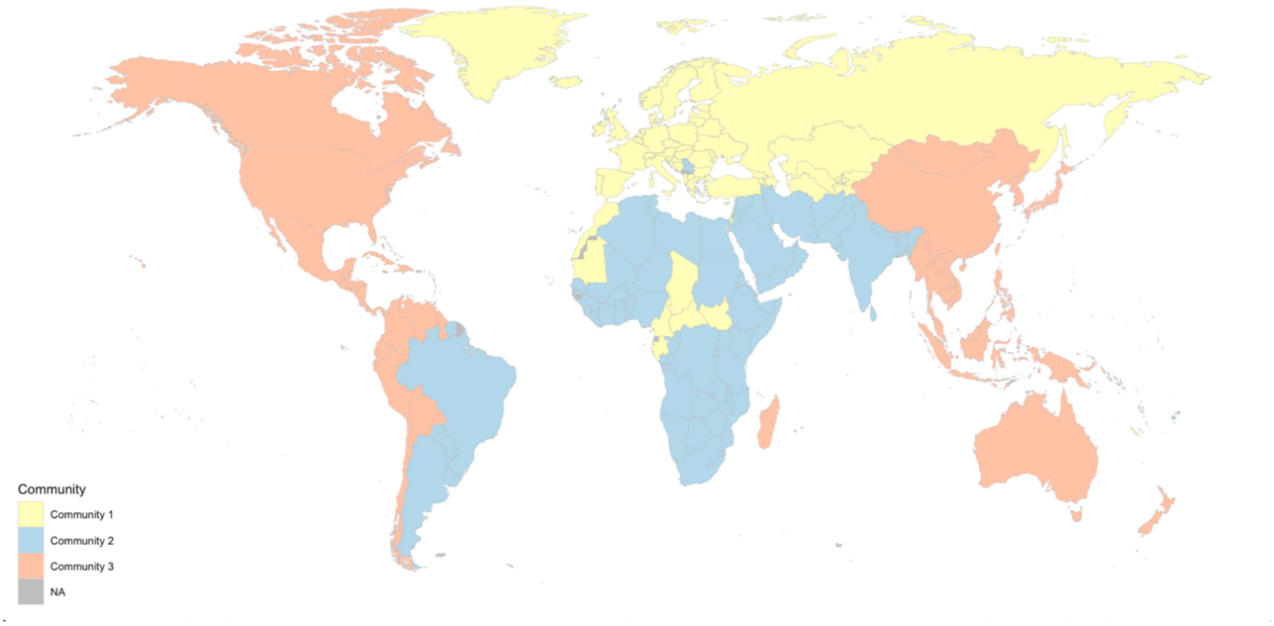

Supplementary Fig. 6 | Regional distribution of communities (Community 1 in yellow, Community 2 in blue, and Community 3 in orange). Map was created by using the R Language, version 4.0.1 (https://www.r-project.org/).

Supplementary Table 1 | Top 20 agricultural NH_3 emission producers, consumers, (net) exporters and importers (Gg).

Top 20	PBE	Top 20	CBE	Top 20	EEE	Top 20	EEI	Top 20	Net export	Top 20	Net import
Mainland China	14648.6	Mainland China	13984.9	Mainland China	1610.2	USA	1563.7	Mainland China	686.3	Japan	995.9
India	6977.4	India	6451.9	USA	960.3	Japan	1003.3	India	533.6	USA	603.5
USA	3699.2	USA	4339.0	India	857.4	Mainland China	924.0	Pakistan	471.9	Hong Kong	490.1
Brazil	2829.2	Brazil	2607.9	Canada	507.8	Germany	742.2	Ethiopia	277.4	UK	480.1
Indonesia	1606.0	Indonesia	1572.9	Pakistan	485.0	UK	556.9	Australia	239.4	Russia	450.4
Pakistan	1320.2	Russia	1347.0	Brazil	388.8	Russia	526.0	Canada	229.0	Germany	376.8
Russia	884.4	Japan	1302.0	Germany	365.5	Hong Kong	490.1	Brazil	225.2	South Korea	316.9
France	869.2	Germany	1219.9	France	364.4	France	450.5	Myanmar	211.0	Italy	187.5
Mexico	842.7	France	965.7	Australia	359.4	Italy	391.7	Belarus	200.0	Saudi Arabia	162.2
Canada	836.9	Mexico	931.9	Ethiopia	280.3	South Korea	330.4	Argentina	177.2	Singapore	112.5
Germany	825.9	UK	925.2	Argentina	230.1	India	323.8	Bangladesh	171.6	Switzerland	103.5
Turkey	781.2	Pakistan	848.6	Spain	226.0	Canada	278.8	New Zealand	161.1	UAE	91.2
Australia	652.8	Turkey	793.9	Myanmar	211.5	Spain	259.4	Egypt	125.4	Malaysia	87.7
Viet Nam	591.9	Italy	751.3	Italy	204.3	Mexico	252.5	Thailand	91.5	France	86.1
Thailand	574.5	Canada	614.4	Belarus	200.2	Netherlands	191.9	Ireland	81.6	Mexico	83.4
Italy	554.8	Spain	577.3	Thailand	191.6	Saudi Arabia	171.4	Chile	79.9	Kuwait	70.0
Iran	549.0	Iran	550.1	Bangladesh	189.1	Brazil	163.5	Viet Nam	78.2	Israel	62.1
Spain	537.8	Viet Nam	514.4	New Zealand	182.2	Malaysia	153.9	Denmark	73.8	Sweden	54.9
Bangladesh	536.8	Hong Kong	501.9	Indonesia	175.6	Indonesia	139.1	Tanzania	71.0	Portugal	53.0
Argentina	519.6	Poland	495.7	Mexico	169.1	Turkey	132.8	Turkmenistan	62.4	Greece	51.4
**Top 20 total**	**40638.2**	**Top 20 total**	**41296.1**	**Top 20 total**	**8158.6**	**Top 20 total**	**9045.9**	**Top 20 total**	**4247.5**	**Top 20 total**	**4918.9**
**Global total**	**52325.0**	**Global total**	**52325.0**	**Global total**	**11839.5**	**Global total**	**11839.5**	**Global total**	**5522.3**	**Global total**	**5522.3**
**Top 20 share**	**77.7%**	**Top 20 share**	**78.9%**	**Top 20 share**	**68.9%**	**Top 20 share**	**76.4%**	**Top 20 share**	**76.9%**	**Top 20 share**	**89.1%**

Supplementary Table 2 | Premature mortality attributable to $PM_{2.5}$ pollution induced by export-driven NH_3 emissions in major exporting countries in 2012 (top 50).

Country	Deaths (× 10^3)	Country	Deaths (× 10^3)
China	26.3	Bulgaria	0.2
India	6.2	Slovakia	0.2
Germany	3.7	Serbia and Montenegro	0.2
Russian Federation	2.7	Austria	0.2
United States	2.1	Brazil	0.2
Poland	2.0	Uzbekistan	0.2
Ukraine	1.6	Nepal	0.2
Italy	1.1	Lithuania	0.2
France	1.1	Switzerland	0.2
Japan	1.0	Iran	0.1
Pakistan	0.9	Moldova	0.1
United Kingdom	0.9	Croatia	0.1
Bangladesh	0.9	Denmark	0.1
Romania	0.8	Greece	0.1
Vietnam	0.7	Myanmar	0.1
Netherlands	0.6	Egypt	0.1
Czech Republic	0.5	Kazakhstan	0.1
Belgium	0.5	Latvia	0.1
North Korea	0.5	Sweden	0.1
Belarus	0.5	Argentina	0.1
Hungary	0.4	Indonesia	0.1
South Korea	0.4	Taiwan China	0.1
Canada	0.3	Thailand	0.1
Spain	0.3	Bosnia and Herzegovina	0.1
Turkey	0.3	Syrian	0.1

Supplementary Table 3 | Top 20 flows of embodied NH_3 emissions (Gg) and health effects (deaths × 10^3).

	Trading pairs	Emissions	Trading pairs	Health effects
1	Mainland China-USA	271	Mainland China-USA	4.4
2	Canada-USA	268	Mainland China-Hong Kong	4.3
3	Mainland China-Hong Kong	264	Mainland China-Japan	4.3
4	Mainland China-Japan	260	Mainland China-South Korea	1.7
5	Belarus-Russia	142	Mainland China-Germany	1.2
6	USA-Japan	140	Ukraine-Russia	1.0
7	USA-Mexico	134	India-USA	0.9
8	India-USA	125	Mainland China-UK	0.9
9	USA-Canada	123	Russia-Mainland China	0.7
10	Mexico-USA	115	Mainland China-France	0.6
11	Mainland China-South Korea	103	Mainland China-Italy	0.5
12	USA-Mainland China	102	Mainland China-Indonesia	0.5
13	Myanmar-Mainland China	99	Mainland China-Canada	0.5
14	Ethiopia-Japan	97	Mainland China-Russia	0.5
15	Pakistan-India	78	Mainland China-Malaysia	0.5
16	Mainland China-Germany	72	India-Mainland China	0.5
17	Ireland-UK	70	Mainland China-Thailand	0.4
18	Pakistan-USA	69	Poland-Germany	0.4
19	Pakistan-Mainland China	65	North Korea-Mainland China	0.4
20	India-Mainland China	62	India-Germany	0.4

Supplementary Table 4 | Health benefits (deaths × 10^3) from different reduction scenarios.

Reduction scenarios	Community 1-EU-CA	Community 2-SWA-AF-SA	Community 3-ESA-NA-OA
Export transfer	0.9	0.2	1.5
Reducing overuse of N in grain crops	1.2	4.5	12.7
Reducing beef consumption by 20%	0.3	0.1	1.4

Supplementary Table 5 | Countries and exported commodities to be adjusted for aggragtion errors.

Country of origin	Exported goods for adjustment
Antigua and Barbuda	Margarine, processed fruits and nuts, raw tobacco
Bangladesh	Raw tobacco, ginger
Belarus	Raw sugar
Central African Republic	Raw cotton, Coffee
Chad	Raw cotton
Côte d'Ivoire	Cocoa beans
Egypt	Citrus fruits
Ethiopia	Coffee
Ghana	Cocoa beans
Guyana	Rice
Madagascar	Vanilla, clove
Mali	Raw cotton
Morocco	Tomatoes, citrus fruit
Myanmar	Dried legumes
Nigeria	Cocoa beans
Papua New Guinea	Palm oil, coffee, cocoa beans
Samoa	Coconut oil
Seychelles	Palm oil
Tanzania	Raw tobacco, coffee, nuts
Uganda	Coffee
Zimbabwe	Raw tobacco
