# Supplementary material for: Mitigation potential of global ammonia emissions and related health impacts in the trade network
Source: Nat Commun. 2021 Nov 5;12:6308. doi: 10.1038/s41467-021-25854-3 (PMC8571346; doi:10.1038/s41467-021-25854-3)
Supplement: Supplementary file 2 — Description of Additional Supplementary Files [file 41467_2021_25854_MOESM2_ESM.pdf]

Description of Additional Supplementary Files

File Name: Supplementary Data 1
Description: Embodied agricultural NH_3 emissions in international exports (Gg).

File Name: Supplementary Data 2
Description: Production-based (PBE) and consumption-based (CBE) agricultural NH_3 emissions.

File Name: Supplementary Data 3
Description: Embodied agricultural NH_3 emissions in international imports (Gg)

File Name: Supplementary Data 4
Description: NH_3 emissions reductions (unit: Gg) achieved through export transfer for the three communities

File Name: Supplementary Data 5
Description: NH_3 emissions reductions (unit: Gg) achieved through import substitution for the three communities

File Name: Supplementary Data 6
Description: Regional information for the MRIO analysis
