# Supplementary material for: Mitigation potential of global ammonia emissions and related health impacts in the trade network
Source: Nat Commun. 2021 Nov 5;12:6308. doi: 10.1038/s41467-021-25854-3 (PMC8571346; doi:10.1038/s41467-021-25854-3)
Supplement: Supplementary file 9 — Reporting Summary [file 41467_2021_25854_MOESM9_ESM.pdf]

nature research

Corresponding author(s): Bo Zhang, Jing Meng

Last updated by author(s): Oct 1, 2021

Reporting Summary

Nature Research wishes to improve the reproducibility of the work that we publish. This form provides structure for consistency and transparency in reporting. For further information on Nature Research policies, see Authors & Referees and the Editorial Policy Checklist .

Statistics

For all statistical analyses, confirm that the following items are present in the figure legend, table legend, main text, or Methods section.

n/a	Confirmed	
x	☐	The exact sample size (*n*) for each experimental group/condition, given as a discrete number and unit of measurement
x	☐	A statement on whether measurements were taken from distinct samples or whether the same sample was measured repeatedly
x	☐	The statistical test(s) used AND whether they are one- or two-sided<br>*Only common tests should be described solely by name; describe more complex techniques in the Methods section.*
x	☐	A description of all covariates tested
x	☐	A description of any assumptions or corrections, such as tests of normality and adjustment for multiple comparisons
x	☐	A full description of the statistical parameters including central tendency (e.g. means) or other basic estimates (e.g. regression coefficient) AND variation (e.g. standard deviation) or associated estimates of uncertainty (e.g. confidence intervals)
x	☐	For null hypothesis testing, the test statistic (e.g. *F*, *t*, *r*) with confidence intervals, effect sizes, degrees of freedom and *P* value noted<br>*Give P values as exact values whenever suitable.*
x	☐	For Bayesian analysis, information on the choice of priors and Markov chain Monte Carlo settings
x	☐	For hierarchical and complex designs, identification of the appropriate level for tests and full reporting of outcomes
x	☐	Estimates of effect sizes (e.g. Cohen's *d*, Pearson's *r*), indicating how they were calculated

Our web collection on statistics for biologists contains articles on many of the points above.

Software and code

Policy information about availability of computer code

Data collection	No software was used.
Data analysis	The GEOS-Chem model code (version 12.0.0) we used is open source (doi: 10.5281/zenodo.1343547). Codes for reproducing the results in this study are available from (https://github.com/KeLi218/NH3_emissions/).

For manuscripts utilizing custom algorithms or software that are central to the research but not yet described in published literature, software must be made available to editors/reviewers. We strongly encourage code deposition in a community repository (e.g. GitHub). See the Nature Research guidelines for submitting code & software for further information.

Data

Policy information about availability of data

All manuscripts must include a data availability statement. This statement should provide the following information, where applicable:

- Accession codes, unique identifiers, or web links for publicly available datasets
- A list of figures that have associated raw data
- A description of any restrictions on data availability

The EDGAR v4.3.2 emission database is from Joint Research Centre, European Commission (https://data.jrc.ec.europa.eu/dataset/jrc-edgar-v432-ap-gridmaps). The global MRIO tables are from https://worldmrio.com/countrywise/. The Eora database is freely accessible from https://www.worldmrio.com/eora/. The United Nations Comtrade Database is available from https://comtrade.un.org/. The population and GDP data are available from the statistical database of the World Bank (https://data.worldbank.org). Country-level baseline mortality for all-age group from the Institute for Health Metrics and Evaluation (http://ghdx.healthdata.org/ihme_data). The area of agricultural land and arable land is obtained from FAO (http://www.fao.org/faostat/en/#data/RL). Average annual temperature and precipitation data are available from the Climatic Research Unit of University of East Anglia (http://www.cru.uea.ac.uk/). The GAINS model is from the International Institute for Applied Systems Analysis (IIASA) (https://gains.iiasa.ac.at/models/gains_models3.htm).

Field-specific reporting

Please select the one below that is the best fit for your research. If you are not sure, read the appropriate sections before making your selection.

☐ Life sciences ☐ Behavioural & social sciences ☒ Ecological, evolutionary & environmental sciences

For a reference copy of the document with all sections, see nature.com/documents/nr-reporting-summary-flat.pdf

Ecological, evolutionary & environmental sciences study design

All studies must disclose on these points even when the disclosure is negative.

Study description	This work estimates trade-induced global agricultural NH3 emissions, consequent PM2.5 formation and related health impacts of the year 2012 in 181 economies, demonstrating large NH3 mitigation potential in international trade and associated benefits.
Research sample	All the databases in this study are publicly accessible. The emission data are from https://data.jrc.ec.europa.eu/dataset/jrc-edgar-v432-ap-gridmaps. The global MRIO tables are from https://worldmrio.com/countrywise/. The population data are obtained from the Gridded Population of the World, version 3 (https://sedac.ciesin.columbia.edu/data/collection/gpw-v3). Country-level baseline mortality for all-age group from the Institute for Health Metrics and Evaluation (http://ghdx.healthdata.org/ihme_data). In this study, premature deaths from ambient PM2.5 exposure include the four leading causes of deaths: ischemic heart disease, chronic obstructive pulmonary disease, cerebrovascular disease, and lung cancer. Then, we estimated the mortality contribution from export-related agricultural NH3 emissions based on an assumption that the contribution of one source to the disease burden of PM2.5 is directly proportional to its share of PM2.5 concentration.
Sampling strategy	The gridded EDGAR emissions have two dominant sectors (livestock and crop) which are the averages over their subsector emissions. The GBD-based air pollutants data for year 2012 at the global scale were adopted according to the combined satellite-based estimates, chemical transport model simulations, and ground measurements. The estimated population data for 2012 were linearly extrapolated from the 2010 and 2011 values. The mortality data for year 2012 at the country-level scale were calculated using the all-age group record. The simulated annul PM2.5 concentrations were calculated using the daily mean concentrations for year 2012.
Data collection	The data used in this study were collected from public website/repository. Bo Zhang downloaded the emission data from https://data.jrc.ec.europa.eu/dataset/jrc-edgar-v432-ap-gridmaps. Ke Li downloaded the population data are obtained from https://sedac.ciesin.columbia.edu/data/collection/gpw-v3, country-level baseline mortality for all-age group from http://ghdx.healthdata.org/ihme_data. Rong Ma downloaded the global MRIO tables from https://worldmrio.com/countrywise/ and the Eora database from https://www.worldmrio.com/eora/. The United Nations Comtrade Database from https://comtrade.un.org/, the area of agricultural land and arable land from http://www.fao.org/faostat/en/#data/RL, and the average annual temperature and precipitation data from http://www.cru.uea.ac.uk/ were also downloaded by Rong Ma.
Timing and spatial scale	The gridded emission, air pollutants, population data for year 2012 at the global scale are used. The mortality data for all-age group for year 2012 at the country-level scale are used. The simulated PM2.5 concentrations for year 2012 at the resolution of 2 degree by 2.5 degree are used.
Data exclusions	No data were excluded in this study.
Reproducibility	This study is conducted by using numerical modeling methods, and can be fully reproducible with the freely-downloaded data.
Randomization	Not applicable. Our study is not experimental and no randomization is needed.
Blinding	Not applicable. Our study is based on numerical model results and hence blinding is not relevant.

Did the study involve field work? ☐ Yes ☒ No

Reporting for specific materials, systems and methods

We require information from authors about some types of materials, experimental systems and methods used in many studies. Here, indicate whether each material, system or method listed is relevant to your study. If you are not sure if a list item applies to your research, read the appropriate section before selecting a response.

Materials & experimental systems

n/a	Involved in the study
☒	☐ Antibodies
☒	☐ Eukaryotic cell lines
☒	☐ Palaeontology
☒	☐ Animals and other organisms
☒	☐ Human research participants
☒	☐ Clinical data

Methods

n/a	Involved in the study
☒	☐ ChIP-seq
☒	☐ Flow cytometry
☒	☐ MRI-based neuroimaging
